# Genome-Wide Association for Morphological and Agronomic Traits in *Phaseolus vulgaris* L. Accessions

**DOI:** 10.3390/plants13182638

**Published:** 2024-09-21

**Authors:** Stephanie Mariel Alves, Giselly Figueiredo Lacanallo, Maria Celeste Gonçalves-Vidigal, Mariana Vaz Bisneta, Andressa Gonçalves Vidigal Rosenberg, Pedro Soares Vidigal Filho

**Affiliations:** 1 Pós-Graduação em Genética e Melhoramento, Universidade Estadual de Maringá, Av. Colombo, 5790, Maringá 87020-900, Brazil; stephaniemarielalves@gmail.com (S.M.A.); marianavazbisneta@gmail.com (M.V.B.); andressa.vidigal@gmail.com (A.G.V.R.); vidigalfilhop@gmail.com (P.S.V.F.); 2 Departamento de Agronomia, Universidade Estadual de Maringá, Av. Colombo, 5790, Maringá 87020-900, Brazil; giselly.fl@gmail.com

**Keywords:** common bean, GWAS, single nucleotide polymorphism

## Abstract

Exploring genetic resources through genomic analyses has emerged as a powerful strategy to develop common bean (*Phaseolus vulgaris* L.) cultivars that are both productive and well-adapted to various environments. This study aimed to identify genomic regions linked to morpho-agronomic traits in Mesoamerican and Andean common bean accessions and to elucidate the proteins potentially involved in these traits. We evaluated 109 common bean accessions over three agricultural years, focusing on traits including the grain yield (YDSD), 100-seed weight (SW), number of seeds per pod (SDPD), number of pods per plant (PDPL), first pod insertion height (FPIH), plant height (PLHT), days to flowering (DF), and days to maturity (DPM). Using multilocus methods such as mrMLM, FASTmrMLM, FASTmrEMMA, ISIS EM-BLASSO, and pLARmEB, we identified 36 significant SNPs across all chromosomes (Pv01 to Pv11). Validating these SNPs and candidate genes in segregating populations is crucial for developing more productive common bean cultivars through marker-assisted selection.

## 1. Introduction

The common bean (*Phaseolus vulgaris* L.) holds a crucial role in human nutrition, both in Brazil and globally [1]. This significance stems from its rich nutritional profile, which includes proteins, micronutrients, and essential minerals [2]. Furthermore, the common bean is widely cultivated across diverse regions worldwide, showcasing its adaptability to a variety of climatic conditions and soil types [3,4].

The genetic variability among common bean cultivars in Brazil serves as an invaluable resource for the development of more productive varieties that are suited to various environmental conditions [5]. Predominantly, the Brazilian germplasm is composed of landrace varieties from the Mesoamerican gene pool, which provides greater genetic diversity compared with the Andean gene pool [6,7,8,9]. The domestication of the common bean fostered genetic diversification, driving physiological and morphological changes. These adaptations, including variations in growth habit, vegetative cycle, photoperiod response, seed shape, color, and size, enabled cultivated plants to thrive in different climates, distinguishing them from their wild progenitors [10,11,12].

Increasing productivity is a central objective in plant breeding programs. Achieving this requires a thorough understanding of the genetic factors that influence productivity and its components. In common bean cultivation, productivity is tied to a variety of morphological, agronomic, and physiological traits. Among these, plant architecture, as well as flowering and maturation periods, play significant roles in the plant’s adaptability and overall productivity [13,14,15].

However, enhancing these traits through traditional breeding methods demands extensive fieldwork, involving evaluations across multiple environments and years. This process is not only costly, but also time-intensive. In this context, the use of molecular markers has emerged as a valuable tool. Molecular markers facilitate the identification of specific genomic regions associated with productivity traits. These regions can be targeted through marker-assisted selection, thereby streamlining the breeding process and making it more efficient and cost-effective [13,16].

Next Generation Sequencing technologies has catalyzed advancements in genomic research, leading to the development of more detailed and dense genetic maps with numerous markers [17]. This genetic information is increasingly being integrated into common bean breeding programs, enabling the application of advanced methodologies such as Genomic Selection (GS), Quantitative Trait Loci (QTL) mapping, and Genome-Wide Association Studies (GWAS) in breeding for complex traits [13,14,15,16,17].

Genetic mapping can be performed using two primary approaches: linkage mapping, which relies on genetic linkage, and association mapping, which is based on linkage disequilibrium (LD). The LD-based approach, commonly known as genome-wide association studies (GWAS), is a powerful tool for identifying genes and QTLs. In GWAS, phenotypic data for a trait of interest are collected from a large group of individuals, along with their genetic information. These data are then analyzed to pinpoint regions of the genome associated with the observed phenotypes. These regions may harbor genes or regulatory elements that are crucial to trait expression, indicating their involvement in the manifestation of the studied trait [18,19].

Genome-wide association studies (GWAS) have proven to be an efficient method for the genetic mapping of quantitative traits and are widely used in the analysis of agronomic characteristics [16,20,21,22,23,24,25]. Furthermore, other studies have focused on identifying genomic associations related to traits such as grain yield and plant phenology in common beans [26,27,28,29]. Most research on genetic variability has focused on germplasm banks, which are vital repositories of genetic diversity. The Germplasm Bank of the Núcleo de Pesquisa Aplicada à Agricultura (Nupagri) at Universidade Estadual de Maringá (UEM) holds traditional cultivars (accessions) from various regions of the country and abroad, alongside elite lines developed for research.

Genome association studies of accessions adapted to specific environments represent an effective approach to investigating quantitative traits. Additionally, multilocus methods have been relatively underexplored in common bean breeding. Thus, this study aims to identify genomic regions associated with morphoagronomic traits in Mesoamerican and Andean common bean accessions from the Nupagri Germplasm Bank.

## 2. Results

### 2.1. Phenotypic Data

**Figure 1:**
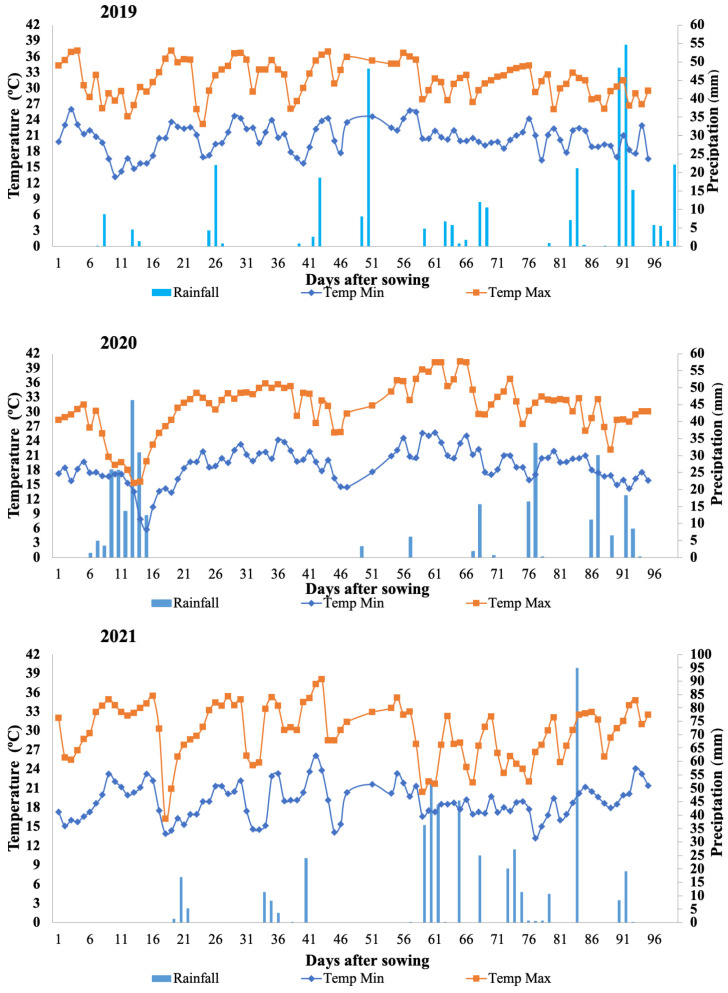
Precipitation (mm) and minimum and maximum temperature (degrees Celsius) in the experiments conducted at the Technical Irrigation Center (CTI) in Maringá, PR, Brazil, in 2019, 2020, and 2021.

The average temperatures were suitable for normal plant development during all experimental periods; the average daily temperature in 2019 was 25.39 °C; in 2020, it was 24.32 °C; and in 2021, it was 23.73 °C. However, the precipitation was insufficient for the optimal development of common bean plants. In 2019, the accumulated rainfall observed was only 346.8 mm, 315.4 mm in 2020, and 470.2 mm in 2021. Thus, in all evaluated years irrigation water management was implemented. In fact, in 2019 and 2021 rainfall was observed during harvesting leading to losses in bean yield (Figure 1).

All traits evaluated in the experiments conducted in 2019, 2020, and 2021 are available in the supplementary file (Table S1). Statistically significant differences and genotype by environment (GxE) interactions were significant across all traits analyzed (Table 1). The average grain yields of the common bean accessions were 990.44 kg ha⁻^1^ in 2019, 1736.26 kg ha⁻^1^ in 2020, and 1093.57 kg ha⁻^1^ in 2021. Notably, the grain yield was highest in 2020, indicating particularly favorable growing conditions during that year, likely due to rainfall at the beginning of the vegetative phase (Figure 1).

The 100-seed weight of the accessions averaged 28.00 g, 27.92 g, and 28.10 g for the years 2019, 2020, and 2021, respectively. Although these variations are minor, they can significantly impact seed quality and commercial value. For the number of seeds per pod and the number of pods per plant, the averages were 3.21, 3.60, and 2.73, and 10.04, 9.67, and 10.42, respectively, across the same years (Table 1). These variations suggest that the plants are adapting to the specific conditions of each environment, presenting opportunities for productivity improvement.

Both the first pod insertion height and plant height showed variability, indicating adaptations in plant architecture to varying environmental conditions. Notably, higher averages were observed in 2020, likely due to the more favorable rainfall conditions. Similarly, the variations in days to flowering and maturity reflected differing responses to the growth cycle among accessions across the years. Although the averages between years showed minimal differences, the significant interaction between genotype and years suggested that certain genotypes may have responded more strongly to specific conditions in a particular year (Table 1). These findings underscore the importance of considering the growth cycle when selecting for more adapted varieties. Overall, the results underscore the phenotypic diversity among the accessions while clearly demonstrating the significant influence of environmental conditions on the traits evaluated. These findings emphasize the critical importance of ongoing research to improve crop performance across diverse environments.

### 2.2. Genome-Wide Association Study (GWAS)

The five ML-GWAS methods identified a total of 36 SNPs (single-nucleotide polymorphisms) associated with the eight evaluated morphoagronomic traits—grain yield (YDSD), 100-seed weight (SW), number of seeds per pod (SDPD), number of pods per plant (PDPL), first pod insertion height (FPIH), plant height (PLHT), number of days to flowering (DF), and number of days to maturity (DPM)—across all chromosomes: Pv01 through Pv11 (Table 2). The largest number of significant SNPs was found on chromosome Pv04, with a total of six SNPs. Chromosomes Pv01, Pv06, and Pv07 each harbored five significant SNPs (Table 2).

#### 2.2.1. SNPs for Yield and Its Components

**Figure 2:**
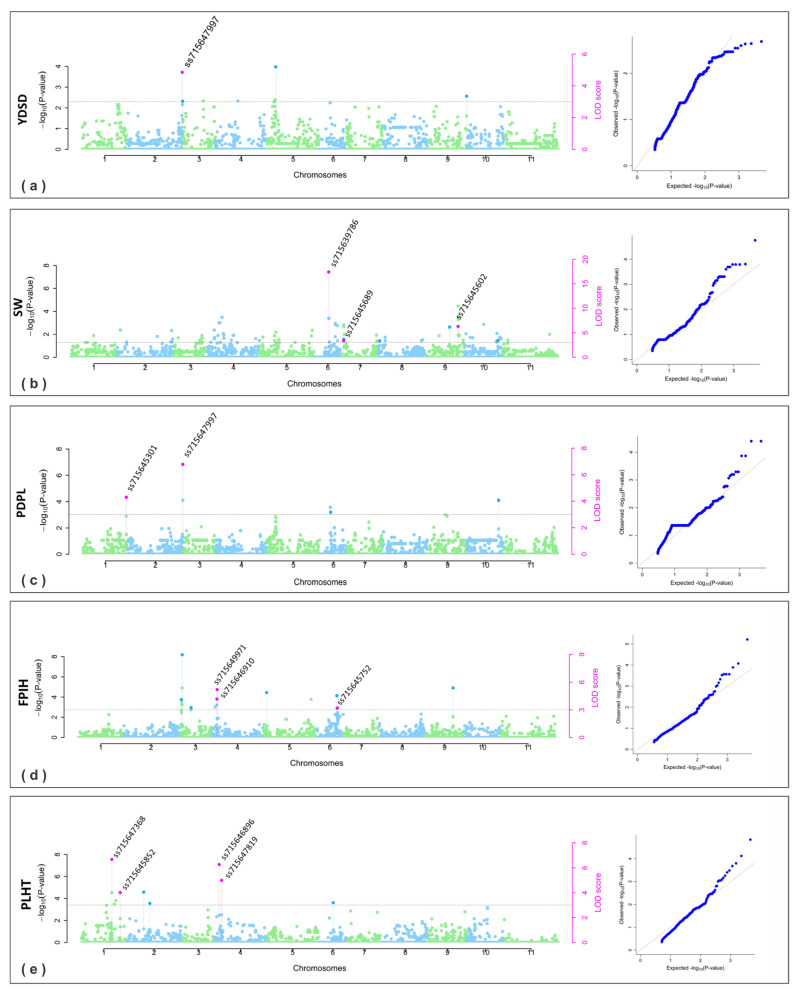
Manhattan and QQ plots obtained in GWAS for the yield and its components and plant architecture: (**a**) YDSD, grain yield (kg ha^−1^); (**b**) SW, 100-seed weight (g); (**c**) PDPL, number of pods per plant; (**d**) FPIH, first pod insertion height (cm); (**e**) PLHT, plant height (cm). The gray threshold line was considered for LOD score ≥ 3. The pink dots indicate the SNPs significantly associated with traits.

A total of 15 significant SNPs were identified for yield and its components, including YDSD, SW, SDPD, and PDPL. Among these, five SNPs associated with YDSD were found on chromosomes Pv02, Pv04, Pv05, Pv07, and Pv08 across the three years analyzed (Table 2). Notably, SNP ss715647997 was observed in the combined analysis, as well as in the year 2019 (Figure 2a). This SNP is located at 48,720,860 bp on chromosome Pv02 within the gene model *Phvul.002G328800* that encodes a serine–threonine protein kinase. It explained 13.38 to 18.32% of the phenotypic variation.

For 100-seed weight (SW), SNPs ss715639786, ss715645689, and ss715645602 were detected on chromosomes Pv06, Pv07, and Pv09, respectively, showing significant associations in the combined analysis and accounting for 4.83% to 57.29% of the phenotypic variation (Figure 2b). SNP ss715645689 was also identified in 2019 and 2020, while SNP ss715645602 was noted in 2021. These SNPs were mapped to genomic positions 22,116,966 bp, 819,750 bp, and 33,444,042 bp, respectively, in the reference genome of common Andean beans [11,30], wherein genes *Phvul.006G105000*, *Phvul.007G011800*, and *Phvul.009G226000* were annotated, encoding for ankyrin repeat-containing protein, methionyl-tRNA synthetase, and aspartyl protease family protein, respectively. 

The SDPD trait was significantly associated in 2020 with SNP ss715645673 on Pv06, explaining 4.92% to 8.24% of the phenotypic variation. This SNP is located at position 27,760,992 bp and is within the genomic region containing the gene model *Phvul.006G166700*, which encodes an abc transporter c family member 11-related protein.

For the PDPL trait, four significant SNPs were identified on chromosomes Pv01, Pv02, Pv10, and Pv11 across all three years analyzed (Table 2). In the combined analysis, SNPs ss715645301 and ss715647997 on chromosomes Pv01 and Pv02, respectively, explained between 8.61% and 43.10% of the phenotypic variation. In the reference genome, close to the ss715645301 marker, the model gene *Phvul.001G264600* is found, which encodes a C2H2-type zinc finger protein. Interestingly, the other SNP ss715647997 is the same marker found to be associated with YDSD, therefore showing a pleiotropic effect.

#### 2.2.2. SNPs for Plant Architecture

Twelve significant SNPs were identified for plant architecture traits, including the first pod insertion height (FPIH) and plant height (PLHT). Among these, six SNPs associated with FPIH were detected on chromosomes Pv03, Pv04, Pv05, Pv06, and Pv09 across the three years analyzed (Table 2), three of them were found in combined analysis. Notably, two SNPs on chromosome Pv04, ss715646910 and ss715649971, were particularly significant. SNP ss715646910 is located in the genomic region containing the gene model *Phvul.004G014500*, which encodes a protein farnesyltransferase subunit beta (FNTB), and explains 9.25% to 10.92% of the phenotypic variation. SNP ss715649971 is located within the model gene *Phvul.004G016000*, which encodes leucine-rich repeat-containing protein, and accounts for 7.02% to 12.51% of the phenotypic variation (Table 2). It is noteworthy that SNP ss715649971 was also observed in the years 2019 and 2021 (Figure 2d). On chromosome Pv06, SNP ss715645752 was found to be associated with FPIH, explaining 6.28% to 21.51% of the phenotypic variation. This SNP is located at genomic position 25,791,849 bp, close to the gene model *Phvul.006G144200*, which encodes serine-threonine protein kinase (Table 2; Figure 2d). This SNP was also observed in 2019 and 2020.

For the plant height (PLHT) trait, six significant SNPs were found to be associated with this trait on chromosomes Pv01, Pv02, Pv04, and Pv07, wherein four were consistently found in combined analysis. On chromosome Pv01, two notable SNPs were identified: ss715647368 and ss715645852. SNP ss715647368 explained 18.12% to 38.65% of the phenotypic variation and was observed in all three years analyzed. It showed pleiotropy, affecting plant height (PLHT), days to flowering (DF), and days to maturity (DPM) (Table 2; Figure 2e). SNP ss715647368 is located at the position 45,894,030 bp close to the gene model *Phvul.001G193200* that encodes a protease s28 pro-x carboxypeptidase-related protein. SNP ss715645852, also on chromosome Pv01, explained 7.10% to 10.16% of the phenotypic variation and was also identified in 2019. This SNP is located at position 49,657,760 bp close to the gene model *Phvul.001G236100* that encodes a poly(a) RNA polymerase gld2 protein.

#### 2.2.3. SNPs for Plant Phenology

**Figure 3:**
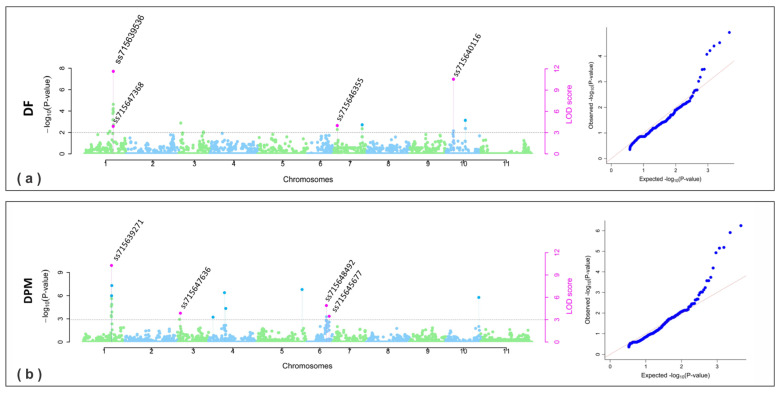
Manhattan and QQ plots obtained in GWAS for phenology: (**a**) DF, number of days for flowering; (**b**) DPM, number of days for maturity. The gray threshold line was considered for LOD score ≥ 3. The pink dots indicate the SNPs significantly associated with traits.

A total of eleven SNPs significantly associated with plant phenology traits, specifically days to flowering (DF) and days to maturity (DPM), were identified across chromosomes Pv01, Pv03, Pv04, Pv06, Pv07, and Pv10 (Table 2). For DF, four SNPs were significant in the combined analysis (Figure 3a). On chromosome Pv01, two notable SNPs were found: ss715647368 (previously described with pleiotropy effect) and ss715639536 at position 46,027,600 bp. SNP ss715647368 explained 12.71% to 27.53% of the phenotypic variation, and SNP ss715639536 accounted for 24.72% to 24.97%. SNP ss715647368 was observed in 2019 and 2020, while ss715639536 was identified in all years analyzed. SNP ss715639536 is positioned near the gene model *Phvul.001G194100*, encoding peroxiredoxin (alkyl hydroperoxide reductase subunit C).

On chromosomes Pv07 and Pv10, SNPs ss715646355 and ss715640116 were identified, explaining phenotypic variations ranging from 1.48% to 5.72% and 14.42% to 56.84%, respectively. Both SNPs were detected across all analyzed years, as well as in the combined analysis (Table 2). SNP ss715646355 is located at the beginning of chromosome 3,770,008 bp in the reference genome of Andean common beans (v1.1) close to the model gene *Phvul.007G046900* that encodes 16S rRNA (uracil(1498)-N(3))-methyltransferase/M(3)U(1498)-specific methyltransferase protein. SNP ss715640116 on Pv10 is positioned at 7,867,881 bp close to *Phvul.010G049800* that encodes a phospholipase A1 (DAD1).

For the trait days to maturity (DPM), nine significant SNPs were identified (Figure 3b); four of them were significant in the combined analysis. The SNP ss715639271 on Pv01 was significantly associated with DPM in the combined analyses and the year 2019, explaining 21.53% to 45.08% of the phenotypic variation. This SNP is located at the end of the chromosome at the position 45,746,595 close to the model gene *Phvul.001G191600*, which encodes beta-fructofuranosidase (E3.2.1.26, sacA). The SNP ss715647636 on chromosome Pv03 was observed in the combined analysis and the year 2019, accounting for 2.44% to 12.81% of the phenotypic variation. This SNP located at 3,963,582 close to *Phvul.003G038400* encodes act domain-containing protein 5.

On chromosome Pv06, two SNPs associated with the days to maturity (DPM) trait were identified in the combined analysis: ss715648492 and ss715645677. SNP ss715648492, located at position 25,619,371 bp, is close to the gene model *Phvul.006G142100*, which encodes armadillo/beta-catenin-like repeat-containing protein. This SNP explained between 5.51% and 16.31% of the phenotypic variation and was detected in the overall analysis, 2019, and 2021 (Figure 3b). SNP ss715645677, located near the gene model *Phvul.006G162500*, which encodes PPR repeat (PPR)//PPR repeat family (PPR_2)//Pentatricopeptide repeat domain (PPR_3), explained phenotypic variation ranging from 3.70% to 5.22%. This SNP was observed in both the combined analysis and specifically in 2019.

## 3. Discussion

### 3.1. Genotype × Environment Interaction

Germplasm banks play a crucial role in conserving and exploring the genetic variability in different bean accessions. The 109 accessions from the Nupagri Germplasm Bank encompass a diverse range of grain types, including Carioca, Black, Jalo, Red, Rosinha, Roxinho, White, and Bolinha. This genetic diversity represents a valuable reservoir of agronomic traits, which are vital for developing more productive cultivars that are resistant to diseases and adapted to various environmental conditions. 

Genotype × environment (G × E) interaction studies are crucial for understanding the phenotypic and genotypic variation in common bean accessions of Mesoamerican and Andean origins. These studies provide insights into how genetic diversity expresses itself under different environmental conditions and help in identifying genotypes that are better adapted to specific environments. Observations of G × E interactions revealed that all evaluated traits exhibited unique behaviors, emphasizing that such interactions can produce varying phenotypic expressions, even for the same genotypes across different environments and years. The presence of G × E interactions is commonly observed in linkage mapping and association mapping studies, where it influences the interactions between QTL × environment and/or SNP × environment [15,27,31].

### 3.2. Common Bean Genomic Regions Associated with Morphological and Agronomic Traits

GWAS has proven to be a valuable tool for exploring genetic diversity, particularly in common bean cultivation. Studies focusing on accessions adapted to specific environments have demonstrated the effectiveness of this approach in investigating quantitative traits, as genomic regions significantly associated with traits across multiple environmental conditions have been identified. Additionally, markers specific to particular environmental conditions have also been discovered [15,27]. Multi-locus methods, which have been relatively underutilized in common bean research, are now gaining prominence for their ability to offer greater statistical power and precision in detecting SNP markers in GWAS [16,25]. This approach is particularly valuable for complex traits that are strongly influenced by environmental factors [32,33]. Multi-locus analyses of accessions from the Nupagri Germplasm Bank have led to significant advances in understanding the genetic control of the evaluated morpho-agronomic traits.

In this study, significant variations were observed among 109 accessions from the Nupagri Germplasm Bank. GWAS revealed genomic regions associated with SNPs for morphoagronomic traits across all chromosomes: Pv01 through Pv11. Similarly, Delfini et al. [15] identified markers related to productivity and its components across all chromosomes in their study of 178 Mesoamerican common bean accessions using ML-GWAS. Different genomic regions were found associated with grain yield (YDSD) and seed weight (SW) across different years. This variability can be attributed to the quantitative nature of these traits, which makes them highly responsive to environmental conditions. Depending on climatic and precipitation patterns, specific regions on the bean chromosomes exhibited varying impacts on these traits. For plant height (PLHT), days to flowering (DF), and days to maturity (DPM), distinct associations were also observed for each trait across years. Nevertheless, the Pv01 regions consistently showed a major effect on controlling these traits throughout the years. In the combined analysis of years; most associations mirrored those identified in one or more individual years, depending on the trait. This approach enriches the findings by revealing consistent patterns and strengthening the overall associations observed.

The GWAS identified significant regions on chromosomes Pv06, Pv07, and Pv09 associated with 100-seed weight (SW). While initial studies mapped 100-seed weight to chromosome Pv07 [34], further research has shown it to be a polygenic quantitative trait distributed across all chromosomes [11,27,34,35,36,37,38]. Many of the identified alleles are linked to domestication processes and ecogeographic factors [11,27,35,36], which aligns with our findings of SW being a quantitative and complex trait among Mesoamerican and Andean accessions.

In the functional annotation of the seed weight (SW) trait, a notable protein identified was an aspartyl protease family protein with similarity to cellulose synthase, which belongs to the CESA gene superfamily. This superfamily, found in *Arabidopsis thaliana* and other seed plants, encodes enzymes essential for cellulose production. The *CESA* genes are divided into groups based on their function and structure, such as *CESA1, CESA2,* and *CESA3*, found in dicotyledonous plants [39,40].

For productivity traits such as grain yield (YDSD) and number of pods per plant (PDPL), a pleiotropic SNP was identified on chromosome Pv02. Similar pleiotropic effects were observed in studies by Delfini et al. [15] and Nkhata et al. [41], who reported comparable findings on chromosome Pv11. These pleiotropic effects suggest that these markers may influence multiple traits in common beans. The genomic region containing this SNP is functionally annotated to a serine/threonine protein kinase, a key player in various cellular processes, including cell cycle regulation, cell signaling, and stress response [42].

For traits related to plant architecture and phenology, including the first pod insertion height (FPIH), plant height (PLHT), days to flowering (DF), and days to maturity (DPM), SNPs were identified on chromosomes Pv01, Pv02, Pv03, Pv04, Pv06, Pv07, Pv09, and Pv10. These traits were also mapped to different chromosomes in Mesoamerican and Andean accessions, which exhibit various growth habits, particularly on chromosomes Pv01, Pv04, Pv06, Pv08, Pv09, Pv10, and Pv11 [14,27,28,43]. The results of this study further evidence the importance of chromosomes Pv01 and Pv10 in controlling bean architecture, as previously described in the literature. Notably, a SNP named ss715647368, located at position 45,894,030 bp on chromosome Pv01, was identified as pleiotropic, indicating a possible relationship between plant architecture and phenology traits (Table 2, Figure 2 and Figure 3). Furthermore, SNP ss715647368 was functionally annotated to a PROTEASE S28 PRO-X carboxypeptidase on chromosome Pv01, which is homologous to the S28 serine carboxypeptidase family protein (*AT5G65760*) in *Arabidopsis thaliana*. These proteins play crucial roles in various biological processes, including growth, development, and the stress response. S28 family carboxypeptidases are particularly vital for regulating protein and peptide maturation, modulating hormonal activity, and enabling plants to adapt to challenging conditions, such as nutrient availability fluctuations and environmental stress [44].

## 4. Materials and Methods

### 4.1. Plant Material

**Figure 4:**
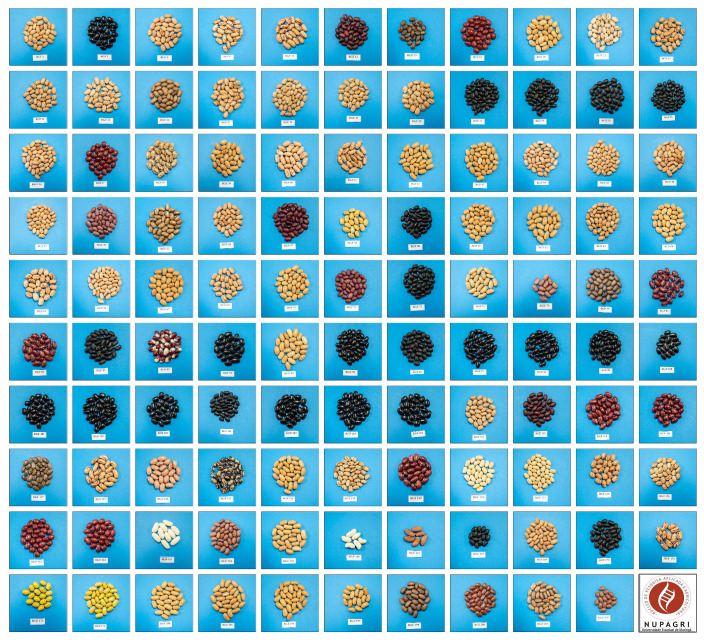
Accessions of common beans from the Nupagri Germplasm Bank (photos taken by UEM/ASC).

This study utilized a total of 109 common bean accessions (55 Mesoamerican and 54 Andean) from the Nupagri Germplasm Bank at the Núcleo de Pesquisa Aplicada à Agricultura, Universidade Estadual de Maringá, Paraná, Brazil. These accessions were collected from farmers in the Brazilian states of Goiás, Mato Grosso do Sul, Minas Gerais, Paraná, and Santa Catarina (Figure 4). Descriptions of the evaluated accessions, such as name, origin, gene pool, and market class, are provided in Table S2.

### 4.2. Phenotyping Evaluation

The experiment was conducted at the Centro de Treinamento em Irrigação (CTI) of the Universidade Estadual de Maringá (UEM) in Maringá, Paraná, Brazil (latitude 23°25′ S and longitude 51°57′ W), during the period from July to November in the agricultural years of 2019, 2020, and 2021. A simple 11 × 11 lattice design was used for the evaluations, covering a total of 109 accessions (Figure 4), along with 12 additional accessions—BRS Pérola, BAT 447, IPR Uirapuru, SEA 5, BRS Esteio, BGF 04, BGF 09, BGF 59, BGF 92, BGF 155, BGF 203, and BGF 204—used to optimize the experimental design.

The experiment included three replications, with plots consisting of 2 m rows spaced 0.5 m apart. The planting density was 12 plants per linear meter, with a border of 4 common bean rows. The plants were fertilized and irrigated according to the specific needs of the crop.

The following morpho-agronomic traits were measured: (a) days to flowering (DF)—the number of days from emergence until 50% of the plants in the plot showed at least one flower; (b) days to physiological maturity (DPM)—the number of days from emergence until 90% of the pods lost their green coloration and began to dry. In the post-harvest phase, the following traits were evaluated: (c) first pod insertion height (FPIH)—the average height, in centimeters, from the soil surface to the first pod insertion; (d) final plant height (PLHT)—the average height, in centimeters, measured from the soil to the tip of the uppermost leaves; (e) number of pods per plant (PDPL)—the total number of pods in the plot divided by the number of plants in the plot; (f) seeds per pod (SDPD)—the average number of seeds in 10 randomly selected pods from each plot; (g) 100-seed weight (SW)—the weight, in grams, of 100 seeds sampled from each plot; and (h) grain yield (YDSD)—measured per plot at 13% moisture and expressed in kg ha^−1^. The crop ontology for agricultural data for the common bean was used as a reference to unify the name of traits (https://cropontology.org/term/CO_335:ROOT (accessed on 4 December 2023)).

### 4.3. Phenotypic Data Analysis

The phenotypic data from each experiment were analyzed using SELEGEN REML/BLUP software (https://www.conectagem.com/softwares (accesses on 7 February 2022)) [45]. Genotypic values were predicted through the best linear unbiased predictor (BLUP) approach, with variance components for random factors estimated via the restricted maximum likelihood (REML) method, following model 52:

y=Xr+Zg+Wb+Ti+e

where y = data vector, r = vector of repetition effects (assumed as fixed) added to the general average, g = vector of genotypic effects (assumed as random), b = vector of block effects (assumed as random), i = vector of genotype × environment interaction effects (random), and e = vector of errors or residues (random). The uppercase letters represent the incidence matrices for the aforementioned effects.

The significance of the model effects was assessed using the Likelihood Ratio Test (LRT), which provided the basis for deviance analysis (ANADEV). Model fit was evaluated by comparing analyses with and without the effects of genotype and genotype × environment interaction. The deviance of the model excluding the specific effect was subtracted from the deviance of the full model, and the resulting value was compared against the chi-square (χ²) distribution with 1 degree of freedom at the 1% and 5% probability levels.

Mathematically, this process is represented as follows:

$$LRT=-2ln\left(\frac{ML\;of\;reduced\;model}{MLV\;of\;complete\;model}\right)$$

where ln denotes the natural logarithm, while ML stands for maximum likelihood estimation. The analyses evaluated the effect of each line on the phenotype, considering fixed effects and random effects to obtain the best linear unbiased estimators (BLUEs) and best linear unbiased predictors (BLUPs), respectively.

### 4.4. Genotyping of the Common Bean Accessions Using BARCBean6K_3 BeadChip

Genomic DNA was extracted from trifoliolate leaves of each accession using the DNeasy Plant Mini Kit (Qiagen, CA, USA) according to the manufacturer’s instructions. The DNA concentration was measured with a Qubit fluorometer, following the manufacturer’s guidelines. The DNA samples were genotyped using 5398 SNP markers on the BARCBean6K_3 Illumina BeadChip, adhering to the Infinium HD Assay Ultra Protocol (Illumina Inc., San Diego, CA, USA). The BeadChip was imaged with the Illumina BeadArray Reader to assess fluorescence intensity [46]. Automatic allele calling for each locus was conducted using Genome Studio Genotyping Module v1.8.4 software (Illumina, San Diego, CA, USA), with all allele calls subsequently verified visually. Data were filtered by excluding SNPs with more than 25% missing calls and minor allele frequencies less than 0.05, resulting in 4633 high-quality SNPs for further analysis.

### 4.5. Genome-Wide Association Study

The GWAS was conducted using adjusted genotypic values for the evaluated traits. These values were derived from REML/BLUP analyses for the years 2019, 2020, and 2021, as well as from the harmonic mean of relative performance of genotypic values (HMRPVG) for the combined analysis of these agricultural years. The population structure with K = 2 for the GWAS was determined using a Bayesian clustering approach with Structure v2.3.4 [47]. The analysis was configured with a burn-in period of 10,000 and 50,000 Markov Chain Monte Carlo (MCMC) iterations, exploring cluster numbers (K) from 2 to 11 across 20 independent runs. Analysis was performed using R software (version 4.0.3) [48] and the multilocus mixed models tool mrMLM.GUI (version 4.0.2) [49], employing five methods: mrMLM [50], FASTmrMLM [51], FASTmrEMMA [52], ISIS EM-BLASSO [53], and pLARmEB [21] for SNP identification. A significance threshold of LOD ≥ 3 was applied across all methods. To reduce false positive associations and enhance statistical power, both population structure and kinship matrix were incorporated into the models. The kinship matrix was calculated using mrMLM.GUI v4.0.2. Model adequacy was evaluated through Q-Q plots, which displayed the observed quantile distribution of –log10 (p) on the y-axis versus the expected quantile distribution of –log10 (p) on the x-axis.

### 4.6. Candidate Gene Identification

Candidate genes within the genomic regions associated with the traits of interest were identified by aligning these regions with the G19833 reference genome v1.0 [11] through the National Center for Biotechnology Information database available at https://www.ncbi.nlm.nih.gov (accessed on 4 April 2023). Functional annotations of each model gene were confirmed using the Phytozome database available at https://phytozome-next.jgi.doe.gov (accessed on 5 April 2023) [30] to predict their potential roles in morphoagronomic traits. This process involved linking the genes to specific biological functions and pathways, thereby aiding in the understanding of their contribution to the traits under investigation.

## 5. Conclusions

This study successfully identified 36 SNPs associated with morphoagronomic traits across all chromosomes (Pv01 to Pv11) in common bean accessions of both Mesoamerican and Andean origins. These SNPs offer valuable insights for improving the productivity and quality of common bean cultivars. Moreover, candidate genes were identified for the most significant SNPs, providing a strong foundation for future research. Ultimately, these findings have the potential to contribute to the development of more productive and resilient common bean varieties, thereby advancing breeding programs and enhancing overall crop performance.

## Figures and Tables

**Table 1:** Traits analyzed in common bean accessions belonging to the Nupagri Germplasm Bank evaluated over three agricultural years, Maringá, PR, Brazil.

Traits	Year	Accessions			*^§^ Gê*	*PEV*
		Average	Interval	LRT (*x*^2^)		
**Yield and its Components**						
Grain yield (kg ha^−1^)	2019	990.44	191.63–1798.61	215.24 **	**	10,221.53
	2020	1736.26	335.15–3723.92	193.41 **		47,059.12
	2021	1093.57	292.21–2469.66	149.20 **		30,490.65
100-seed weight (g)	2019	28.00	15.22–49.73	611.54 **	**	0.99
	2020	27.92	16.21–54.63	565.88 **		1.27
	2021	28.10	14.15–44.50	421.76 **		2.32
Number of seeds per pod	2019	3.21	2.12–4.54	135.70 **	**	0.05
	2020	3.60	1.91–5.99	154.82 **		0.09
	2021	2.73	2.05–3.52	38.85 **		0.05
Number of pods per plant	2019	10.04	4.34–15.14	119.59 **	**	1.37
	2020	9.67	3.50–17.35	119.90 **		1.32
	2021	10.42	5.45–21.00	60.21 **		3.81
**Architecture**						
First pod insertion height (cm)	2019	13.34	11.32–18.14	150.65 **	**	0.30
	2020	13.53	11.21–21.71	152.08 **		0.56
	2021	13.14	11.19–16.01	51.08 **		0.44
Plant height (cm)	2019	49.00	37.56–58.63	309.44 **	**	1.17
	2020	50.12	36.17–60.80	492.35 **		0.68
	2021	47.88	37.77–58.08	134.13 **		3.33
**Phenology**						
Number of days for flowering	2019	42.70	30.99–60.99	1208.73 **	**	0.06
	2020	42.26	31.73–60.96	925.23 **		0.22
	2021	42.71	30.00–60.00	1281.12 **		0.0009
Number of days for maturity	2019	81.46	73.65–89.59	360.75 **	**	1.03
	2020	81.49	73.57–91.30	292.75 **		1.32
	2021	80.89	73.28–87.69	263.45 **		2.35

** = significant by the chi-square test with one degree of freedom: at 1% (6.63); LRT (*x*^2^) = likelihood-ratio test (chi-square); ^§^ *Gê* = interaction between genotype treatments and years; *PEV* = variance in the error of prediction for genotypic values.

**Table 2:** SNPs associated with yield and its components, plant architecture, and phenology identified using different methods in common bean accessions from the Nupagri Germplasm Bank, Maringá, PR, Brazil.

^§^ Traits	Year	Chr	Position (pb)	SNP	^#^ LOD Score	−Log_10_ *P*	^¥^ SNP Effect	^¶^ R^2^ (%)	Methods
YDSD	2019	2	48,720,860	ss715647997	4.55	5.33	−147.00~−124.64	12.43~17.29	1, 5
	2020	4	25,245,150	ss715650918	4.72	5.50	−513.05~−361.05	9.81~16.55	1, 2
		7	39,295,293	ss715640487	5.35~6.10	6.16~6.94	−303.83~−235.68	11.69~17.70	1, 2, 4, 5
		8	55,278,106	ss715639359	3.99	4.74	148.49~238.74	5.73~12.37	1, 2
	2021	5	5,399,253	ss715648672	4.63	5.41	−299.91~−150.73	12.70~12.96	3, 4
	C *	2	48,720,860	ss715647997	4.85	5.64	−203.83~−175.25	13.38~18.32	1, 4
SW	2019	7	819,750	ss715645689	3.21~9.19	3.92~10.11	−7.55~−2.99	13.48~21.51	2, 3, 4
		10	24,598,176	ss715641543	3.46~4.95	4.18~5.74	−6.90~−3.79	10.13~21.71	3, 4
	2020	2	3,118,774	ss715639502	3.41~9.10	4.13~10.01	−6.48~−3.38	2.08~29.97	1, 3, 4
		5	40,128,137	ss715646697	5.01~5.90	5.81~6.73	2.75~6.47	11.28~15.64	2, 3
		7	819,750	ss715645689	5.75~10.15	6.57~11.09	−7.30~−3.45	17.83~25.23	2, 3, 4, 5
	2021	9	33,444,042	ss715645602	6.41~7.89	7.26~8.78	−3.03~−2.03	5.30~11.75	1, 4, 5
	C *	6	22,116,966	ss715639786	4.49~30.63	5.26~31.54	−6.21~−3.52	18.43~57.29	4, 5
		7	819,750	ss715645689	3.10~3.38	3.80~4.10	−9.49~−2.34	8.15~33.45	2, 3, 4
		9	33,444,042	ss715645602	5.17~7.42	5.98~8.29	−2.13~−1.96	4.83~5.74	4, 5
SDPD	2020	6	27,760,992	ss715645673	3.77~4.00	4.51~4.76	−0.35~−0.32	4.92~8.24	1, 5
PDPL	2019	1	51,819,821	ss715645301	3.22~15.14	3.93~16.16	0.80~1.77	10.11~43.39	1, 2, 3, 4, 5
		2	48,720,860	ss715647997	4.80~8.15	5.59~9.04	−0.97~−0.88	8.83~10.74	1, 4, 5
		10	39,797,018	ss715646330	3.18~4.09	3.89~4.85	−1.03~−0.61	2.92~8.13	1, 2
	2021	10	39,797,018	ss715646330	3.13~5.64	3.83~6.46	−2.38~−0.95	5.07~15.24	1, 2, 3, 4, 5
		11	48,780,038	ss715650748	6.69~6.86	7.54~7.72	−1.59~−1.12	9.46~19.15	1, 4
PDPL	C *	1	51,819,821	ss715645301	3,54~14,89	4.27~15.91	0.95~1.92	12.22~43.10	1, 2, 3, 4, 5
		2	48,720,860	ss715647997	4.56~6.89	5.34~7.76	−1.04~−0.99	8.61~9.36	1, 4, 5
FPIH	2019	3	4,083,079	ss715647965	4.86~7.70	5.65~8.59	−0.46~−0.30	4.32~12.85	4, 5
		4	1,627,690	ss715649971	4.03~9.59	4.78~10.52	0.36~0.63	7.00~21.68	1, 2, 4, 5
		6	25,791,849	ss715645752	3.09~6.38	3.79~7.23	−0.83~−0.56	9.98~21.74	1, 2
		9	31,832,898	ss715647626	4.23~5.39	4.99~6.21	0.42~0.50	7.12~10.12	2, 4, 5
	2020	5	40,141,917	ss715646699	3.53~4.09	4.25~4.85	−0.95~−0.88	7.20~8.47	1, 2
		6	25,791,849	ss715645752	4.45~5.82	5.22~6.65	−2.31~−0.94	14.47~19.87	1, 2, 3, 5
	2021	4	1,627,690	ss715649971	3.33~4.37	4.05~5.14	0.29~0.73	8.98~23.46	1, 2, 3, 4
	C *	4	1,503,482	ss715646910	3.93~4.41	4.67~5.18	−1.05~−0.59	9.25~10.92	3, 4
		4	1,627,690	ss715649971	4.02~6.35	4.77~7.20	0.39~0.52	7.02~12.51	1, 2
		6	25,791,849	ss715645752	3.04~6.30	3.74~7.15	−0.89~−0.59	6.28~21.51	1, 2, 4
PLHT	2019	1	45,894,030	ss715647368	5.24~15.56	6.05~16.59	−6.00~−2.04	18.04~43.65	1, 2, 3, 4, 5
		1	49,657,760	ss715645852	3.90~4.06	4.65~4.82	−1.25~−1.58	7.43~10.31	1, 2, 5
		4	1,224,240	ss715646896	6.17	7.00	−1.69~−2.25	14.20~21.93	1, 2
		4	1,982,297	ss715647819	4.95	5.75	1.53~2.02	9.91~15.00	1, 2
	2020	1	45,894,030	ss715647368	4.49~12.03	5.27~13.01	−6.18~−3.09	29.53~33.98	2, 3, 4, 5
	2021	1	45,894,030	ss715647368	4.58~8.20	5.35~9.10	−5.17~−1.94	20.00~30.53	1, 2, 3, 4, 5
		2	45,319,921	ss715645964	4.73~5.02	5.51~5.82	−1.82~−1.28	7.57~15.23	1, 2, 4
		7	3,648,568	ss715646353	3.25	3.96	−1.26~−0.82	3.45~8.13	1, 2
	C *	1	45,894,030	ss715647368	5.29~15.61	6.10~16.64	−6.16~−2.11	18.12~38.65	1, 2, 3, 4, 5
		1	49,657,760	ss715645852	3.81~4.01	4.55~4.76	−1.62~−1.26	7.10~10.16	1, 2, 5
		4	1,224,240	ss715646896	5.11~6.26	5.91~7.10	−2.33~−1.37	8.59~22.18	1, 2, 4
		4	1,982,297	ss715647819	3.74~4.99	4.48~5.78	1.14~2.08	5.04~15.05	1, 2, 4
DF	2019	1	45,894,030	ss715647368	3.83	4.57	−3.75~−2.56	12.80~27.40	1, 2
		1	46,027,600	ss715639536	11.42~11.94	12.38~12.91	−3.68~−3.53	24.52~26.65	4, 5
		7	3,770,008	ss715646355	3.02~3.98	3.71~4.73	−2.19~−1.13	1.49~5.60	1, 2, 5
		10	7,867,881	ss715640116	10.39~12.48	11.35~13.46	−3.35~−3.98	23.79~33.55	1, 2, 5
	2020	1	45,894,030	ss715647368	3.51	4.24	−3.90~−2.53	10.82~25.80	1, 2
		1	46,027,600	ss715639536	7.80~11.41	8.69~12.37	−3.41~−3.22	17.46~19.89	4, 5
		7	3,770,008	ss715646355	4.13	4.88	−2.37~−1.74	3.07~5.72	1, 2
		10	7,867,881	ss715640116	11.04	12.00	3.69~4.39	25.10~35.50	1, 2
	2021	1	46,027,600	ss715639536	10.57~16.56	11.52~17.60	−3.93~−3.30	23.26~33.10	4, 5
		7	3,770,008	ss715646355	3.48~3.69	4.20~4.42	−1.33~−1.28	1.93~2.27	1, 2
		7	48,520,131	ss715648580	4.10~5.64	4.85~6.47	2.50~3.32	3.01~5.30	4, 5
		10	7,867,881	ss715640116	7.94~9.19	8.83~10.11	2.50~5.17	14.42~56.84	1, 2, 5
	C *	1	45,894,030	ss715647368	3.89	4.63	−3.79~−2.57	12.71~27.53	1, 2
		1	46,027,600	ss715639536	11.14~12.15	12.11~13.13	−3.59~−3.57	24.72~24.97	4, 5
		7	3,770,008	ss715646355	3.02~3.99	3.72~4.75	−2.19~−1.13	1.48~5.33	1, 2, 5
		10	7,867,881	ss715640116	10.54~12.65	11.48~13.64	3.57~4.02	23.78~33.67	1, 2, 5
DPM	2019	1	45,746,595	ss715639271	7.39~7.70	8.27~8.58	−2.80~−2.22	21.53~22.62	1, 2
		1	46,027,600	ss715639536	3.42~7.59	4.14~8.47	−2.31~−2.24	22.66~24.25	4, 5
DPM	2019	3	3,963,582	ss715647636	3.03~4.84	3.73~5.63	0.83~1.39	2.44~6.83	4, 5
		4	4,497,307	ss715649528	4.93~7.78	5.73~8.67	2.52~2.79	29.33~32.58	1, 2
		6	25,619,371	ss715648492	5.99~6.07	6.82~6.90	−1.99~−1.64	12.34~16.31	1, 2
		6	27,370,471	ss715645677	3.27~5.77	3.98~5.60	1.05~2.15	5.01~5.22	3, 4
	2020	1	46,027,600	ss715639536	3.75~7.15	4.49~8.01	−2.46~−1.58	13.04~22.07	1, 2, 5
		3	4,083,079	ss715647965	4.33~5.07	5.09~5.87	−2.65~−1.84	15.28~24.25	1, 2
		10	39,577,266	ss715646318	4.35~5.16	5.12~5.96	2.12~2.81	20.14~26.89	1, 2
	2021	1	45,894,030	ss715647368	4.48~6.05	5.25~6.88	−3.47~−1.74	8.44~10.71	1, 2, 3, 4
		4	4,497,307	ss715649528	4.90~16.14	5.69~17.18	2.97~5.95	30.35~51.06	1, 2, 3, 4
		6	25,619,371	ss715648492	3.27~3.80	3.99~4.54	−1.83~−1.31	5.50~9.45	1, 2
	C *	1	45,746,595	ss715639271	8.22~12.34	9.11~13.33	−3.18~−2.26	22.92~45.08	1, 2
		3	3,963,582	ss715647636	3.31~4.33	4.02~5.20	1.18~1.95	4.69~12.81	1, 5
		6	25,619,371	ss715648492	3.51~6.28	4.23~7.13	−1.63~−1.12	5.51~11.86	1, 2
		6	27,370,471	ss715645677	3.22~3.70	3.92~4.43	0.93~2.19	3.70~5.15	3, 4

^§^ Traits: YDSD, grain yield (kg ha^−1^); SW, 100-seed weight (g); SDPD, number of seeds per pod; PDPL, number of pod per plant; FPIH, first pod insertion height (cm); PLHT, plant height (cm); DF, number of days for flowering; DPM, number of days for maturity; C *, combined analysis of years using the harmonic mean of relative performance of genotypic values (HMRPVG); Chr, chromosome; ^#^ LOD value ≥ 3; ^¥^ SNP effect, Single-nucleotide polymorphism effect on evaluated traits found through GWAS using multilocus mixed models; ^¶^ indicates the percentage of phenotypic variation explained by each SNP; methods: 1, mrMLM; 2, FASTmrMLM; 3, FASTmrEMMA; 4, pLARmEB; 5, ISIS EM-BLASSO.

## Data Availability

All data are presented within the article.

## Supplementary Materials

The following supporting information can be downloaded at: https://www.mdpi.com/article/10.3390/plants13182638/s1, Table S1. Phenotypic data from common beans accessions from Nupagri-UEM germplasm bank evaluated during the years of 2019, 2020, and 2021. Table S2 Accessions from Nupagri-UEM germoplasm bank, gene pool, origin, and market class evaluated in the present study.
