# Supplementary material for: Genome-Wide Association for Morphological and Agronomic Traits in Phaseolus vulgaris L. Accessions
Source: Plants (Basel). 2024 Sep 21;13(18):2638. doi: 10.3390/plants13182638 (PMC11435040; doi:10.3390/plants13182638)
Supplement: Supplementary file 1 [file plants-13-02638-s001.zip › Table S1.pdf]

Table S1. Phenotypic data from common beans accessions from Nupagri-UEM germplasm bank evaluated during the years of 2019, 2020 and 2021.

Year	Plot	Acessions	Replication	Block	Interaction	Observation /Plot	YDSD	SW	SDPD	PDPL	FPIH	PLHT	DF	DPM
1	1	1	1	1	11	1	809.28	22.30	2.76	9.20	13.50	39.67	39.00	73.00
1	2	3	1	1	13	1	1263.03	32.00	3.32	10.72	12.10	50.33	34.00	73.00
1	3	5	1	1	15	1	1095.89	21.35	3.19	14.18	12.40	47.33	40.00	73.00
1	4	6	1	1	16	1	891.24	39.10	2.37	8.97	11.90	44.83	32.00	80.00
1	5	11	1	1	111	1	698.83	40.90	2.33	7.53	12.20	37.50	33.00	73.00
1	6	12	1	1	112	1	1162.14	21.75	4.08	10.28	14.50	57.67	46.00	88.00
1	7	13	1	1	113	1	92.73	17.75	3.79	2.83	15.45	51.17	61.00	86.00
1	8	15	1	1	115	1	899.11	31.60	3.36	9.23	13.40	52.10	40.00	73.00
1	9	20	1	1	120	1	981.55	38.80	3.70	6.28	11.80	52.33	40.00	80.00
1	10	22	1	1	122	1	1008.78	23.80	3.55	11.51	12.30	50.33	38.00	73.00
1	11	23	1	1	123	1	2150.16	32.55	3.75	15.58	14.30	61.60	48.00	88.00
1	12	24	1	2	124	1	1013.06	25.89	3.95	12.05	13.40	48.83	50.00	88.00
1	13	25	1	2	125	1	675.50	24.65	2.66	9.76	16.00	51.50	40.00	88.00
1	14	26	1	2	126	1	915.06	23.15	2.98	11.57	13.20	54.50	51.00	80.00
1	15	27	1	2	127	1	657.92	24.55	2.93	7.06	13.50	51.67	50.00	90.00
1	16	28	1	2	128	1	1203.22	22.65	4.28	13.05	13.70	50.50	50.00	88.00
1	17	29	1	2	129	1	671.22	20.90	4.47	11.32	12.20	50.25	50.00	88.00
1	18	30	1	2	130	1	1085.00	31.00	3.18	11.38	17.80	51.67	50.00	90.00
1	19	32	1	2	132	1	1004.11	19.95	3.47	10.83	13.65	50.17	50.00	88.00
1	20	33	1	2	133	1	1478.17	23.05	4.66	11.93	14.00	54.67	41.00	84.00
1	21	34	1	2	134	1	547.56	21.30	2.61	12.40	18.10	54.33	47.00	90.00
1	22	35	1	2	135	1	932.94	16.40	3.55	15.11	15.50	58.33	51.00	88.00
1	23	36	1	3	136	1	666.94	19.50	3.64	10.72	11.30	43.00	44.00	80.00
1	24	37	1	3	137	1	1463.39	35.30	3.13	9.13	13.60	53.67	40.00	77.00
1	25	38	1	3	138	1	483.39	40.30	2.95	5.42	9.60	38.28	31.00	75.00
1	26	39	1	3	139	1	955.11	36.65	3.21	11.42	12.80	41.50	32.00	77.00
1	27	40	1	3	140	1	1158.50	44.45	2.73	7.57	11.60	47.11	34.00	79.00
1	28	41	1	3	141	1	1090.83	40.05	2.52	8.59	11.60	40.67	33.00	75.00
1	29	42	1	3	142	1	1198.94	37.45	3.42	5.87	13.40	45.67	38.00	77.00
1	30	43	1	3	143	1	813.17	34.70	2.69	5.11	9.70	40.33	35.00	77.00
1	31	44	1	3	144	1	533.17	18.25	3.98	7.47	11.70	47.34	40.00	78.00

Year	Plot	Acessions	Replication	Block	Interaction	Observation /Plot	YDSD	SW	SDPD	PDPL	FPIH	PLHT	DF	DPM
1	32	45	1	3	145	1	428.17	18.80	4.49	9.49	14.73	47.58	51.00	80.00
1	33	46	1	3	146	1	1123.89	19.95	3.67	11.42	12.40	44.16	40.00	77.00
1	34	47	1	4	147	1	170.30	13.90	3.03	4.08	10.75	45.25	51.00	80.00
1	35	49	1	4	149	1	1010.33	20.85	3.85	9.86	16.00	51.00	51.00	88.00
1	36	52	1	4	152	1	970.28	26.40	3.42	11.80	14.30	43.50	40.00	80.00
1	37	56	1	4	156	1	1433.44	24.85	4.00	15.78	14.10	48.33	40.00	80.00
1	38	57	1	4	157	1	738.50	23.05	3.23	9.91	13.90	49.00	51.00	84.00
1	39	58	1	4	158	1	256.28	24.30	2.04	5.41	19.65	50.83	49.00	90.00
1	40	60	1	4	160	1	1007.61	17.60	3.52	12.71	14.30	54.00	51.00	88.00
1	41	61	1	4	161	1	1078.00	35.05	3.17	7.06	12.60	52.33	34.00	73.00
1	42	62	1	4	162	1	865.28	39.75	2.88	6.16	12.70	46.83	31.00	77.00
1	43	63	1	4	163	1	653.33	19.33	4.24	7.78	12.35	50.50	49.00	80.00
1	44	64	1	4	164	1	1140.61	33.35	3.62	6.58	10.80	50.00	40.00	77.00
1	45	65	1	5	165	1	1317.55	37.55	3.35	8.87	14.20	48.00	40.00	80.00
1	46	66	1	5	166	1	826.02	18.95	3.84	12.75	13.70	48.33	50.00	88.00
1	47	67	1	5	167	1	882.39	35.20	3.00	7.00	12.60	39.50	31.00	77.00
1	48	69	1	5	169	1	968.33	23.50	3.72	12.96	13.10	54.17	49.00	84.00
1	49	70	1	5	170	1	1379.00	32.90	3.50	10.00	14.00	49.00	40.00	80.00
1	50	71	1	5	171	1	811.61	22.20	3.44	10.35	12.70	47.00	45.00	80.00
1	51	72	1	5	172	1	1277.89	18.80	4.90	22.28	13.53	49.67	49.00	84.00
1	52	73	1	5	173	1	765.72	35.85	2.92	9.61	11.78	37.50	31.00	73.00
1	53	74	1	5	174	1	343.78	17.85	3.40	10.99	11.90	40.67	41.00	80.00
1	54	75	1	5	175	1	1029.19	23.90	3.14	14.97	15.55	45.16	42.00	78.00
1	55	82	1	5	182	1	1442.00	40.80	3.37	9.26	14.60	53.67	40.00	77.00
1	56	83	1	6	183	1	1055.44	41.20	2.80	8.86	12.90	43.67	34.00	78.00
1	57	85	1	6	185	1	502.77	21.90	3.26	9.54	19.50	54.33	51.00	90.00
1	58	89	1	6	189	1	668.50	40.50	2.60	6.81	11.60	40.83	35.00	80.00
1	59	91	1	6	191	1	1205.17	36.15	3.04	9.80	14.00	48.83	35.00	77.00
1	60	93	1	6	193	1	1078.78	37.60	3.52	5.52	11.80	43.17	34.00	77.00
1	61	94	1	6	194	1	791.39	20.70	2.97	9.28	16.60	50.17	51.00	88.00
1	62	95	1	6	195	1	1114.67	13.90	3.61	7.36	12.00	51.50	50.00	84.00
1	63	97	1	6	197	1	1166.28	35.35	2.77	7.33	11.60	49.83	38.00	77.00

Year	Plot	Acessions	Replication	Block	Interaction	Observation /Plot	YDSD	SW	SDPD	PDPL	FPIH	PLHT	DF	DPM
1	64	98	1	6	198	1	1069.05	28.70	3.64	7.13	12.90	45.83	40.00	73.00
1	65	99	1	6	199	1	1546.22	32.60	4.26	8.71	11.50	49.67	39.00	73.00
1	66	100	1	6	1100	1	662.01	18.95	2.94	7.75	13.45	54.67	48.00	80.00
1	67	101	1	7	1101	1	1116.89	33.35	3.18	9.06	12.40	45.50	40.00	77.00
1	68	102	1	7	1102	1	1477.78	33.60	3.61	5.53	11.80	46.50	35.00	73.00
1	69	104	1	7	1104	1	1045.72	36.45	2.76	7.07	12.30	51.83	40.00	80.00
1	70	105	1	7	1105	1	869.17	20.00	3.91	9.69	13.70	53.50	51.00	80.00
1	71	107	1	7	1107	1	814.72	31.95	3.51	6.59	12.80	54.22	35.00	73.00
1	72	110	1	7	1110	1	1160.05	39.35	3.38	8.04	11.30	50.83	39.00	80.00
1	73	111	1	7	1111	1	1421.39	35.85	3.57	11.10	12.10	48.11	39.00	73.00
1	74	112	1	7	1112	1	1207.89	23.30	3.57	14.07	12.80	51.67	44.00	80.00
1	75	113	1	7	1113	1	1753.89	22.40	4.14	14.18	14.70	58.50	49.00	80.00
1	76	114	1	7	1114	1	891.72	33.05	2.62	7.98	11.70	48.94	34.00	84.00
1	77	115	1	7	1115	1	836.89	36.70	2.74	6.85	10.80	41.33	34.00	77.00
1	78	117	1	8	1117	1	1041.83	18.55	3.04	16.33	18.43	50.00	51.00	90.00
1	79	119	1	8	1119	1	825.22	40.90	2.27	9.25	10.90	40.17	34.00	73.00
1	80	120	1	8	1120	1	1021.22	27.45	3.07	13.67	12.80	48.17	40.00	80.00
1	81	121	1	8	1121	1	941.89	19.85	3.75	21.01	11.90	52.50	40.00	84.00
1	82	128	1	8	1128	1	976.11	35.85	2.72	7.55	13.80	52.17	40.00	77.00
1	83	138	1	8	1138	1	883.24	21.25	3.80	11.30	13.30	51.33	50.00	84.00
1	84	139	1	8	1139	1	762.61	34.90	2.40	5.47	12.20	44.00	34.00	80.00
1	85	144	1	8	1144	1	483.93	18.23	2.05	7.81	13.20	52.00	50.00	84.00
1	86	153	1	8	1153	1	885.57	18.90	5.28	15.38	13.90	51.33	50.00	84.00
1	87	154	1	8	1154	1	606.93	36.40	2.82	7.36	14.50	47.50	34.00	74.00
1	88	156	1	8	1156	1	1664.05	22.70	4.15	14.32	14.10	50.67	50.00	84.00
1	89	161	1	9	1161	1	929.48	37.90	2.74	11.77	15.20	51.50	44.00	80.00
1	90	162	1	9	1162	1	343.39	32.55	2.12	4.93	12.50	46.17	34.00	84.00
1	91	163	1	9	1163	1	281.01	38.94	2.33	3.94	9.17	45.67	34.00	80.00
1	92	164	1	9	1164	1	784.00	21.40	2.92	8.13	12.00	47.12	38.00	84.00
1	93	165	1	9	1165	1	829.89	40.40	3.04	5.72	12.80	55.17	40.00	80.00
1	94	166	1	9	1166	1	112.58	32.03	1.90	3.25	12.20	44.50	34.00	88.00
1	95	167	1	9	1167	1	392.39	57.25	2.50	3.94	12.85	39.83	35.00	88.00

Year	Plot	Acessions	Replication	Block	Interaction	Observation /Plot	YDSD	SW	SDPD	PDPL	FPIH	PLHT	DF	DPM
1	96	168	1	9	1168	1	1142.59	21.10	3.48	19.17	12.88	55.17	49.00	84.00
1	97	169	1	9	1169	1	1036.00	20.75	3.39	16.92	11.50	48.00	45.00	80.00
1	98	170	1	9	1170	1	1164.33	23.10	4.04	11.15	15.50	50.00	51.00	88.00
1	99	172	1	9	1172	1	1057.00	42.15	2.83	7.69	10.80	43.78	34.00	74.00
1	100	174	1	10	1174	1	1029.97	32.45	2.93	5.66	13.90	51.50	46.00	80.00
1	101	175	1	10	1175	1	704.67	33.25	3.37	13.23	14.05	50.67	40.00	80.00
1	102	188	1	10	1188	1	811.22	37.00	3.61	4.58	10.30	49.83	39.00	74.00
1	103	191	1	10	1191	1	787.11	38.20	3.02	6.46	13.80	50.00	41.00	84.00
1	104	194	1	10	1194	1	1000.96	38.40	2.88	6.03	12.80	54.28	40.00	74.00
1	105	197	1	10	1197	1	1075.28	37.10	2.77	6.69	12.60	49.00	40.00	80.00
1	106	199	1	10	1199	1	748.24	24.35	2.83	9.96	12.50	45.17	46.00	76.00
1	107	200	1	10	1200	1	1630.41	18.20	6.13	14.79	13.80	51.33	51.00	84.00
1	108	201	1	10	1201	1	728.78	25.40	3.71	12.91	13.80	49.72	51.00	89.00
1	109	205	1	10	1205	1	991.18	18.50	3.73	12.53	13.93	50.62	51.00	84.00
1	110	500	1	10	1500	1	659.17	21.05	3.26	8.21	13.50	53.67	51.00	80.00
1	111	501	1	11	1501	1	1136.29	22.45	2.64	9.90	14.83	51.67	51.00	88.00
1	112	502	1	11	1502	1	1615.05	21.35	5.15	15.91	13.60	56.83	50.00	88.00
1	113	503	1	11	1503	1	1527.17	26.10	4.24	10.93	14.90	50.67	44.00	80.00
1	114	504	1	11	1504	1	1229.62	24.10	3.96	16.28	14.33	54.00	49.00	88.00
1	115	505	1	11	1505	1	384.54	19.00	3.00	7.80	15.65	56.17	51.00	84.00
1	116	506	1	11	1506	1	736.17	37.45	2.44	6.99	13.90	51.67	40.00	80.00
1	117	507	1	11	1507	1	513.25	17.55	4.20	9.54	13.05	51.00	51.00	84.00
1	118	508	1	11	1508	1	908.05	32.85	3.15	7.50	13.90	50.05	36.00	73.00
1	119	509	1	11	1509	1	914.42	20.45	2.48	11.95	15.38	46.66	50.00	90.00
1	120	510	1	11	1510	1	1254.55	34.75	3.32	10.38	12.70	51.11	39.00	80.00
1	121	511	1	11	1511	1	495.10	13.00	3.29	7.81	15.10	50.00	51.00	88.00
1	122	1	2	12	11	1	747.83	21.90	2.95	10.89	13.00	40.17	39.00	77.00
1	123	3	2	12	13	1	1431.50	33.10	4.42	11.14	13.00	51.00	34.00	77.00
1	124	5	2	12	15	1	858.67	23.10	3.76	8.55	11.30	44.67	41.00	80.00
1	125	6	2	12	16	1	765.72	38.70	2.71	11.71	14.30	41.83	32.00	77.00
1	126	11	2	12	111	1	810.83	40.30	2.64	6.09	11.80	37.00	33.00	77.00
1	127	12	2	12	112	1	1326.89	22.60	4.22	12.81	14.50	56.83	46.00	80.00

Year	Plot	Acessions	Replication	Block	Interaction	Observation /Plot	YDSD	SW	SDPD	PDPL	FPIH	PLHT	DF	DPM
1	128	13	2	12	113	1	102.86	19.96	2.66	4.35	15.70	55.50	61.00	91.00
1	129	15	2	12	115	1	1485.17	33.80	3.54	10.15	13.80	51.50	40.00	77.00
1	130	20	2	12	120	1	679.39	30.55	3.08	7.00	14.00	52.00	40.00	80.00
1	131	22	2	12	122	1	1162.78	22.35	3.01	17.55	12.00	47.67	38.00	80.00
1	132	23	2	12	123	1	1959.22	29.45	3.42	11.70	17.70	58.84	51.00	88.00
1	133	24	2	13	124	1	1957.66	24.20	5.41	14.43	12.30	49.00	50.00	88.00
1	134	25	2	13	125	1	758.72	23.55	2.94	11.65	15.00	53.33	40.00	88.00
1	135	26	2	13	126	1	1734.44	26.05	4.18	13.47	13.30	56.83	49.00	80.00
1	136	27	2	13	127	1	1260.00	30.75	2.66	14.35	16.90	51.67	50.00	90.00
1	137	28	2	13	128	1	1176.39	23.20	5.07	17.28	13.25	49.50	50.00	88.00
1	138	29	2	13	129	1	640.11	21.30	3.70	22.34	12.05	46.67	50.00	88.00
1	139	30	2	13	130	1	1527.55	30.40	3.45	11.22	14.70	52.50	50.00	90.00
1	140	32	2	13	132	1	1345.17	19.90	4.33	18.45	13.10	51.33	50.00	88.00
1	141	33	2	13	133	1	1183.78	22.20	4.53	15.04	14.40	57.83	41.00	84.00
1	142	34	2	13	134	1	638.47	17.25	2.14	18.81	17.80	53.17	50.00	90.00
1	143	35	2	13	135	1	1008.39	15.15	3.95	12.18	13.20	56.50	51.00	89.00
1	144	36	2	14	136	1	907.67	17.95	3.66	12.83	12.80	38.33	44.00	80.00
1	145	37	2	14	137	1	1041.05	33.20	2.99	8.44	11.67	50.34	40.00	77.00
1	146	38	2	14	138	1	1100.94	39.10	3.29	10.78	12.00	38.50	31.00	77.00
1	147	39	2	14	139	1	1192.72	32.65	3.14	7.58	10.70	40.92	32.00	77.00
1	148	40	2	14	140	1	1070.61	40.60	3.00	8.78	11.70	46.17	34.00	77.00
1	149	41	2	14	141	1	1462.61	38.75	2.92	9.19	10.80	39.83	34.00	77.00
1	150	42	2	14	142	1	1008.00	36.45	3.48	6.54	14.60	51.50	40.00	77.00
1	151	43	2	14	143	1	1131.67	30.40	3.41	6.99	11.50	39.00	35.00	77.00
1	152	44	2	14	144	1	776.61	19.20	4.39	12.67	12.70	43.50	40.00	84.00
1	153	45	2	14	145	1	437.50	18.40	3.68	9.72	14.05	49.00	51.00	88.00
1	154	46	2	14	146	1	1368.11	16.90	3.92	13.93	12.70	42.67	40.00	77.00
1	155	47	2	15	147	1	284.28	16.55	4.05	4.37	12.92	46.33	51.00	80.00
1	156	49	2	15	149	1	886.28	20.05	3.33	13.02	18.10	50.83	51.00	88.00
1	157	52	2	15	152	1	1538.05	26.10	4.58	12.99	12.50	46.50	40.00	84.00
1	158	56	2	15	156	1	1312.34	19.85	3.90	10.08	11.40	47.17	40.00	80.00
1	159	57	2	15	157	1	943.44	23.30	3.06	9.37	13.30	52.83	51.00	88.00

Year	Plot	Acessions	Replication	Block	Interaction	Observation /Plot	YDSD	SW	SDPD	PDPL	FPIH	PLHT	DF	DPM
1	160	58	2	15	158	1	256.28	24.80	1.72	8.22	17.30	49.00	48.00	90.00
1	161	60	2	15	160	1	739.27	16.80	2.98	9.25	13.48	51.33	49.00	88.00
1	162	61	2	15	161	1	1523.28	32.20	3.79	8.85	13.70	47.33	34.00	77.00
1	163	62	2	15	162	1	765.72	40.00	2.75	8.24	12.40	46.00	31.00	77.00
1	164	63	2	15	163	1	929.83	20.70	4.44	11.65	10.80	47.83	49.00	80.00
1	165	64	2	15	164	1	1254.94	32.70	3.09	8.00	12.60	49.17	40.00	77.00
1	166	65	2	16	165	1	1351.39	37.55	3.22	10.47	15.10	51.00	40.00	80.00
1	167	66	2	16	166	1	1029.78	17.50	3.75	14.22	12.40	49.17	50.00	88.00
1	168	67	2	16	167	1	1142.17	34.70	2.42	10.65	10.50	38.83	31.00	77.00
1	169	69	2	16	169	1	1246.93	20.95	4.66	7.58	13.40	47.83	51.00	88.00
1	170	70	2	16	170	1	1434.61	32.45	3.48	11.52	13.70	48.83	39.00	80.00
1	171	71	2	16	171	1	1020.83	22.30	3.68	11.20	12.60	50.83	45.00	80.00
1	172	72	2	16	172	1	1213.83	16.43	4.68	11.45	11.51	51.83	51.00	80.00
1	173	73	2	16	173	1	1273.22	39.70	3.28	8.35	13.00	42.67	31.00	77.00
1	174	74	2	16	174	1	510.56	19.64	2.79	8.84	10.42	41.33	41.00	80.00
1	175	75	2	16	175	1	952.78	24.05	3.74	12.31	13.90	42.50	42.00	80.00
1	176	82	2	16	182	1	1913.88	39.10	2.79	15.88	13.20	52.83	40.00	84.00
1	177	83	2	17	183	1	1561.00	42.30	3.04	8.35	15.30	47.17	34.00	80.00
1	178	85	2	17	185	1	895.61	23.45	3.67	11.09	18.60	51.17	51.00	90.00
1	179	89	2	17	189	1	1031.02	40.30	2.62	11.07	11.60	41.84	35.00	77.00
1	180	91	2	17	191	1	1141.39	35.95	3.35	10.63	11.70	48.67	35.00	77.00
1	181	93	2	17	193	1	1205.94	37.00	3.19	9.83	10.80	43.94	34.00	77.00
1	182	94	2	17	194	1	823.67	17.20	4.22	11.64	15.20	51.00	51.00	84.00
1	183	95	2	17	195	1	2189.05	20.65	5.03	23.05	13.40	50.00	50.00	89.00
1	184	97	2	17	197	1	1406.61	38.00	2.99	8.38	12.60	48.67	39.00	77.00
1	185	98	2	17	198	1	1617.78	32.25	3.31	12.41	13.30	42.17	39.00	73.00
1	186	99	2	17	199	1	1193.34	29.40	2.94	6.63	11.50	49.11	39.00	73.00
1	187	100	2	17	1100	1	1167.99	21.55	2.59	11.03	13.51	51.33	51.00	80.00
1	188	101	2	18	1101	1	1354.89	33.50	3.74	7.54	12.30	47.33	39.00	77.00
1	189	102	2	18	1102	1	1210.77	32.65	3.99	7.20	12.80	45.00	35.00	73.00
1	190	104	2	18	1104	1	1083.05	33.70	3.21	7.28	12.60	57.33	40.00	77.00
1	191	105	2	18	1105	1	1243.78	18.75	4.53	8.92	12.80	53.67	51.00	84.00

Year	Plot	Acessions	Replication	Block	Interaction	Observation /Plot	YDSD	SW	SDPD	PDPL	FPIH	PLHT	DF	DPM
1	192	107	2	18	1107	1	964.44	32.10	3.52	6.93	11.90	54.00	35.00	73.00
1	193	110	2	18	1110	1	1252.61	33.45	3.65	7.33	12.90	45.83	39.00	77.00
1	194	111	2	18	1111	1	1100.55	34.30	3.26	7.07	14.60	46.83	39.00	73.00
1	195	112	2	18	1112	1	1501.93	23.45	2.88	14.28	12.90	47.17	44.00	80.00
1	196	113	2	18	1113	1	2129.04	20.20	3.99	11.70	12.35	58.50	51.00	80.00
1	197	114	2	18	1114	1	1007.61	34.10	3.36	6.16	11.50	50.16	34.00	84.00
1	198	115	2	18	1115	1	1249.50	40.10	3.19	7.42	13.30	45.67	34.00	77.00
1	199	117	2	19	1117	1	709.72	17.70	3.96	10.95	18.20	51.33	51.00	90.00
1	200	119	2	19	1119	1	1269.33	42.45	2.90	13.66	12.10	40.83	34.00	77.00
1	201	120	2	19	1120	1	1804.83	31.70	3.37	14.90	13.00	48.67	40.00	77.00
1	202	121	2	19	1121	1	662.28	20.00	2.60	11.79	13.50	49.67	40.00	84.00
1	203	128	2	19	1128	1	1327.67	33.40	3.81	8.44	12.00	50.50	40.00	77.00
1	204	138	2	19	1138	1	1044.55	21.90	3.86	10.46	15.60	53.00	51.00	84.00
1	205	139	2	19	1139	1	540.17	36.40	2.82	9.38	13.00	40.33	34.00	77.00
1	206	144	2	19	1144	1	551.76	18.35	3.14	4.81	13.20	50.33	50.00	80.00
1	207	153	2	19	1153	1	1366.17	18.20	5.34	13.98	15.60	48.00	51.00	88.00
1	208	154	2	19	1154	1	752.91	32.60	3.27	8.09	12.70	48.60	34.00	74.00
1	209	156	2	19	1156	1	1401.94	20.15	4.63	11.10	12.15	47.17	51.00	84.00
1	210	161	2	20	1161	1	567.78	36.90	2.69	6.36	13.80	49.00	44.00	80.00
1	211	162	2	20	1162	1	701.55	37.20	2.20	5.61	14.60	42.33	34.00	80.00
1	212	163	2	20	1163	1	279.61	39.00	1.74	7.19	12.28	48.00	34.00	80.00
1	213	164	2	20	1164	1	978.83	22.95	3.19	10.68	14.70	47.50	39.00	80.00
1	214	165	2	20	1165	1	471.33	35.45	2.48	5.36	13.30	55.83	40.00	80.00
1	215	166	2	20	1166	1	142.29	36.66	1.95	5.80	12.02	42.17	34.00	88.00
1	216	167	2	20	1167	1	317.33	44.65	2.05	2.71	13.20	38.50	34.00	88.00
1	217	168	2	20	1168	1	1107.17	20.45	3.15	13.67	13.90	53.33	49.00	84.00
1	218	169	2	20	1169	1	1543.54	20.65	3.60	13.31	11.30	50.83	46.00	80.00
1	219	170	2	20	1170	1	1452.11	21.50	4.57	13.28	14.60	52.33	50.00	88.00
1	220	172	2	20	1172	1	1132.05	40.85	2.95	9.22	12.70	44.23	34.00	77.00
1	221	174	2	21	1174	1	1152.37	31.25	3.38	7.12	14.80	51.83	45.00	80.00
1	222	175	2	21	1175	1	596.56	32.80	3.40	11.06	13.30	51.67	40.00	80.00
1	223	188	2	21	1188	1	1289.55	34.95	3.84	7.44	13.10	46.95	39.00	77.00

Year	Plot	Acessions	Replication	Block	Interaction	Observation /Plot	YDSD	SW	SDPD	PDPL	FPIH	PLHT	DF	DPM
1	224	191	2	21	1191	1	756.19	33.70	2.47	5.90	12.53	50.33	40.00	84.00
1	225	194	2	21	1194	1	1383.67	40.50	3.73	7.15	12.80	49.78	40.00	77.00
1	226	197	2	21	1197	1	1049.61	34.20	3.16	7.03	14.10	52.17	40.00	80.00
1	227	199	2	21	1199	1	978.83	23.15	4.08	11.57	12.70	45.00	46.00	82.00
1	228	200	2	21	1200	1	1905.16	16.70	3.52	15.08	13.00	46.50	51.00	88.00
1	229	201	2	21	1201	1	1074.50	23.95	3.23	17.84	16.70	48.17	51.00	88.00
1	230	205	2	21	1205	1	874.61	16.90	3.36	18.64	13.50	49.50	51.00	88.00
1	231	500	2	21	1500	1	595.31	17.45	2.72	6.05	11.20	51.00	51.00	80.00
1	232	501	2	22	1501	1	1268.17	24.25	3.05	17.27	16.70	54.00	51.00	88.00
1	233	502	2	22	1502	1	1730.94	22.50	4.99	14.77	13.40	56.28	51.00	88.00
1	234	503	2	22	1503	1	1638.78	27.20	3.80	10.86	14.70	54.17	44.00	80.00
1	235	504	2	22	1504	1	1983.72	25.55	5.14	14.81	15.40	54.50	51.00	88.00
1	236	505	2	22	1505	1	513.72	18.60	3.25	14.46	15.70	55.33	51.00	84.00
1	237	506	2	22	1506	1	1307.05	39.30	2.92	8.11	13.20	48.50	40.00	80.00
1	238	507	2	22	1507	1	1109.89	17.60	3.83	16.69	13.00	46.17	51.00	88.00
1	239	508	2	22	1508	1	1232.39	32.75	3.51	8.28	12.10	49.67	35.00	77.00
1	240	509	2	22	1509	1	982.64	22.50	3.68	11.75	16.60	51.33	50.00	90.00
1	241	510	2	22	1510	1	1435.00	33.80	4.18	8.28	11.40	50.00	40.00	80.00
1	242	511	2	22	1511	1	605.14	14.90	3.77	10.08	12.13	46.17	51.00	88.00
1	243	1	3	23	11	1	718.28	20.90	2.90	10.75	11.40	40.17	39.00	74.00
1	244	3	3	23	13	1	1275.17	36.40	3.14	12.26	12.20	51.83	34.00	78.00
1	245	5	3	23	15	1	969.93	18.30	2.88	6.83	11.80	44.83	42.00	84.00
1	246	6	3	23	16	1	738.50	38.00	2.38	7.02	11.20	43.78	32.00	77.00
1	247	11	3	23	111	1	742.39	40.15	2.48	6.40	12.45	36.17	33.00	77.00
1	248	12	3	23	112	1	1228.50	22.35	4.02	13.13	15.00	54.83	46.00	80.00
1	249	13	3	23	113	1	99.94	16.75	2.41	5.75	16.85	54.83	61.00	90.00
1	250	15	3	23	115	1	1190.15	30.30	2.36	9.63	12.10	50.33	40.00	80.00
1	251	20	3	23	120	1	758.33	33.05	2.69	6.43	12.30	52.33	40.00	80.00
1	252	22	3	23	122	1	1476.61	26.95	3.82	12.43	11.35	46.94	38.00	78.00
1	253	23	3	23	123	1	1571.26	30.55	3.31	9.88	15.60	57.00	49.00	88.00
1	254	24	3	24	124	1	1485.03	23.55	4.65	12.29	14.00	47.33	50.00	84.00
1	255	25	3	24	125	1	572.44	22.60	2.66	10.62	14.80	51.00	40.00	84.00

Year	Plot	Acessions	Replication	Block	Interaction	Observation /Plot	YDSD	SW	SDPD	PDPL	FPIH	PLHT	DF	DPM
1	256	26	3	24	126	1	1107.94	23.65	3.00	15.63	14.20	53.50	50.00	80.00
1	257	27	3	24	127	1	511.39	29.45	2.60	6.93	14.20	58.00	50.00	90.00
1	258	28	3	24	128	1	714.78	19.35	3.88	17.55	15.80	47.50	50.00	88.00
1	259	29	3	24	129	1	698.44	22.40	4.29	10.12	11.90	51.00	50.00	88.00
1	260	30	3	24	130	1	1220.45	30.45	3.54	9.40	15.60	53.33	50.00	90.00
1	261	32	3	24	132	1	1316.00	18.30	4.29	14.90	13.90	51.67	50.00	88.00
1	262	33	3	24	133	1	1084.88	19.40	2.92	11.42	15.90	57.33	40.00	84.00
1	263	34	3	24	134	1	310.96	15.70	2.01	14.15	17.50	60.67	49.00	90.00
1	264	35	3	24	135	1	895.61	15.10	3.82	21.32	14.65	55.17	51.00	88.00
1	265	36	3	25	136	1	548.25	17.15	3.44	12.05	14.60	40.55	44.00	84.00
1	266	37	3	25	137	1	1284.89	35.60	3.31	8.76	13.70	47.33	40.00	77.00
1	267	38	3	25	138	1	809.28	39.45	2.63	11.46	11.20	39.45	31.00	77.00
1	268	39	3	25	139	1	1185.33	33.10	3.65	10.95	13.20	41.28	32.00	77.00
1	269	40	3	25	140	1	992.83	39.25	3.43	8.28	13.30	46.33	34.00	83.00
1	270	41	3	25	141	1	1080.72	39.90	2.49	11.76	12.10	38.50	34.00	77.00
1	271	42	3	25	142	1	927.11	39.55	3.41	4.98	14.30	46.00	39.00	74.00
1	272	43	3	25	143	1	1102.50	33.20	3.09	10.55	11.78	39.50	35.00	77.00
1	273	44	3	25	144	1	561.17	19.50	3.53	11.90	13.00	49.50	40.00	80.00
1	274	45	3	25	145	1	431.53	16.80	3.73	7.54	12.83	50.33	51.00	84.00
1	275	46	3	25	146	1	1208.40	20.10	4.23	13.16	13.68	48.33	40.00	77.00
1	276	47	3	26	147	1	207.01	16.60	2.51	4.18	12.28	50.00	51.00	78.00
1	277	49	3	26	149	1	852.08	19.10	3.50	11.38	16.00	43.83	51.00	88.00
1	278	52	3	26	152	1	1082.28	26.30	3.76	15.88	11.80	41.83	40.00	84.00
1	279	56	3	26	156	1	855.17	22.35	3.85	7.73	13.60	50.33	40.00	80.00
1	280	57	3	26	157	1	800.45	24.35	3.05	10.00	12.40	51.33	51.00	80.00
1	281	58	3	26	158	1	251.26	22.68	1.93	7.06	18.95	50.67	48.00	90.00
1	282	60	3	26	160	1	682.11	17.95	2.03	11.59	13.30	52.50	50.00	88.00
1	283	61	3	26	161	1	1024.72	32.55	3.42	8.93	13.40	50.33	34.00	78.00
1	284	62	3	26	162	1	1108.72	39.95	2.87	8.03	12.90	45.50	31.00	77.00
1	285	63	3	26	163	1	751.72	18.00	3.86	7.49	14.20	50.33	49.00	80.00
1	286	64	3	26	164	1	1164.24	27.65	3.01	5.30	14.10	49.50	40.00	77.00
1	287	65	3	27	165	1	1221.75	32.65	2.74	9.57	14.00	50.67	40.00	84.00

Year	Plot	Acessions	Replication	Block	Interaction	Observation /Plot	YDSD	SW	SDPD	PDPL	FPIH	PLHT	DF	DPM
1	288	66	3	27	166	1	789.32	19.34	3.34	9.73	13.20	49.83	50.00	88.00
1	289	67	3	27	167	1	639.72	30.25	2.29	7.97	11.50	40.50	31.00	77.00
1	290	69	3	27	169	1	1139.99	23.88	4.11	11.23	14.20	54.66	50.00	84.00
1	291	70	3	27	170	1	1095.50	33.90	3.38	7.89	13.15	51.17	40.00	80.00
1	292	71	3	27	171	1	873.36	20.60	3.14	9.64	9.80	53.50	45.00	77.00
1	293	72	3	27	172	1	1039.58	14.54	3.16	10.98	13.05	52.33	50.00	80.00
1	294	73	3	27	173	1	872.28	35.65	2.68	7.30	11.30	39.67	32.00	77.00
1	295	74	3	27	174	1	516.83	20.35	3.60	7.65	11.80	39.00	41.00	80.00
1	296	75	3	27	175	1	1205.55	25.75	4.17	10.76	12.20	46.67	42.00	74.00
1	297	82	3	27	182	1	1160.05	37.50	2.69	9.13	14.90	53.33	40.00	77.00
1	298	83	3	28	183	1	1189.80	39.25	2.93	6.70	13.40	42.00	34.00	77.00
1	299	85	3	28	185	1	728.35	22.88	3.38	8.01	18.94	54.67	51.00	90.00
1	300	89	3	28	189	1	662.16	39.70	2.09	8.57	10.80	43.50	35.00	80.00
1	301	91	3	28	191	1	798.78	35.70	2.74	8.76	13.35	50.00	35.00	80.00
1	302	93	3	28	193	1	814.72	39.45	3.19	5.81	13.30	41.50	34.00	77.00
1	303	94	3	28	194	1	601.96	18.60	3.86	12.23	13.30	52.89	51.00	88.00
1	304	95	3	28	195	1	1334.90	16.35	3.73	8.55	12.00	53.33	50.00	88.00
1	305	97	3	28	197	1	1194.08	38.70	2.15	6.25	11.80	53.17	39.00	84.00
1	306	98	3	28	198	1	1235.89	32.15	3.47	10.81	12.00	46.17	40.00	74.00
1	307	99	3	28	199	1	1055.44	31.65	3.08	8.61	10.30	49.17	40.00	74.00
1	308	100	3	28	1100	1	883.85	20.45	3.15	9.98	13.35	53.17	49.00	84.00
1	309	101	3	29	1101	1	1373.55	32.75	3.93	8.54	13.50	46.67	40.00	77.00
1	310	102	3	29	1102	1	960.73	32.50	3.25	5.46	11.10	48.67	35.00	78.00
1	311	104	3	29	1104	1	1185.91	35.30	2.37	8.21	13.40	48.22	40.00	80.00
1	312	105	3	29	1105	1	994.31	18.15	4.21	9.68	13.70	54.22	51.00	84.00
1	313	107	3	29	1107	1	727.61	32.60	3.38	7.78	11.50	50.17	35.00	74.00
1	314	110	3	29	1110	1	1155.78	34.60	3.39	8.33	12.20	44.17	39.00	77.00
1	315	111	3	29	1111	1	1311.72	35.85	3.35	7.85	10.90	50.50	39.00	74.00
1	316	112	3	29	1112	1	1233.17	25.35	3.46	11.50	13.10	54.33	44.00	80.00
1	317	113	3	29	1113	1	1453.28	21.30	4.48	11.91	14.80	53.94	50.00	80.00
1	318	114	3	29	1114	1	939.65	35.10	2.43	6.82	15.80	44.33	34.00	84.00
1	319	115	3	29	1115	1	997.58	38.35	2.98	7.10	12.40	44.17	34.00	84.00

Year	Plot	Acessions	Replication	Block	Interaction	Observation /Plot	YDSD	SW	SDPD	PDPL	FPIH	PLHT	DF	DPM
1	320	117	3	30	1117	1	907.16	17.15	3.09	13.01	19.67	50.17	51.00	90.00
1	321	119	3	30	1119	1	1345.40	40.35	2.40	11.75	10.90	42.33	34.00	77.00
1	322	120	3	30	1120	1	976.50	29.30	4.00	15.80	11.30	48.00	40.00	74.00
1	323	121	3	30	1121	1	560.98	19.38	2.83	16.16	12.47	50.00	40.00	84.00
1	324	128	3	30	1128	1	1055.83	37.60	3.40	7.11	13.20	47.67	40.00	80.00
1	325	138	3	30	1138	1	1164.16	20.80	3.92	8.83	12.40	51.66	51.00	88.00
1	326	139	3	30	1139	1	982.33	41.00	3.20	6.19	12.85	43.67	34.00	84.00
1	327	144	3	30	1144	1	792.94	20.55	3.03	9.47	14.90	53.00	50.00	84.00
1	328	153	3	30	1153	1	1050.81	19.70	3.72	11.46	13.20	54.67	51.00	84.00
1	329	154	3	30	1154	1	669.51	31.65	2.99	9.58	12.23	48.83	34.00	74.00
1	330	156	3	30	1156	1	1526.15	18.85	4.24	13.06	12.20	50.00	50.00	88.00
1	331	161	3	31	1161	1	936.06	31.00	2.55	9.12	13.38	51.00	44.00	80.00
1	332	162	3	31	1162	1	453.83	34.08	1.95	4.95	12.70	48.17	34.00	80.00
1	333	163	3	31	1163	1	369.44	44.65	1.92	4.47	12.30	50.67	34.00	80.00
1	334	164	3	31	1164	1	768.09	21.20	3.42	10.20	12.40	43.83	38.00	80.00
1	335	165	3	31	1165	1	570.30	35.33	2.84	5.40	12.61	50.89	40.00	80.00
1	336	166	3	31	1166	1	157.93	35.90	1.79	3.50	11.44	42.95	34.00	88.00
1	337	167	3	31	1167	1	239.87	48.23	1.78	2.58	13.48	39.33	34.00	88.00
1	338	168	3	31	1168	1	681.92	21.30	2.75	13.85	14.30	51.33	49.00	84.00
1	339	169	3	31	1169	1	1101.72	21.95	3.32	10.46	13.10	49.67	46.00	80.00
1	340	170	3	31	1170	1	901.05	21.45	4.57	11.65	14.85	54.95	51.00	88.00
1	341	172	3	31	1172	1	1025.89	37.85	2.47	9.11	13.15	44.33	34.00	74.00
1	342	174	3	32	1174	1	1103.28	32.85	3.18	8.73	14.95	50.92	46.00	80.00
1	343	175	3	32	1175	1	589.16	29.70	2.16	15.68	13.20	47.35	40.00	80.00
1	344	188	3	32	1188	1	1573.83	37.60	4.03	7.98	12.40	45.83	39.00	74.00
1	345	191	3	32	1191	1	795.31	34.90	2.51	5.97	13.30	51.83	40.00	84.00
1	346	194	3	32	1194	1	1205.55	37.30	3.78	7.87	13.00	55.50	40.00	74.00
1	347	197	3	32	1197	1	911.23	31.10	2.59	3.95	10.60	52.67	40.00	80.00
1	348	199	3	32	1199	1	818.61	25.35	4.90	10.54	12.40	47.33	46.00	78.00
1	349	200	3	32	1200	1	1085.59	19.70	2.79	13.29	14.20	50.00	51.00	88.00
1	350	201	3	32	1201	1	661.11	22.85	2.89	12.74	13.30	52.50	51.00	88.00
1	351	205	3	32	1205	1	766.11	18.35	3.26	14.90	13.40	49.83	51.00	88.00

Year	Plot	Acessions	Replication	Block	Interaction	Observation /Plot	YDSD	SW	SDPD	PDPL	FPIH	PLHT	DF	DPM
1	352	500	3	32	1500	1	493.81	17.50	3.08	5.49	12.90	52.30	51.00	80.00
1	353	501	3	33	1501	1	798.39	24.00	2.08	17.45	14.60	53.33	51.00	88.00
1	354	502	3	33	1502	1	1466.91	20.95	3.94	9.42	13.35	57.83	50.00	88.00
1	355	503	3	33	1503	1	1246.49	24.85	3.13	8.95	14.60	52.12	44.00	82.00
1	356	504	3	33	1504	1	1239.39	22.25	4.14	11.25	14.30	54.83	50.00	88.00
1	357	505	3	33	1505	1	449.75	17.50	3.42	9.95	18.80	57.50	51.00	84.00
1	358	506	3	33	1506	1	867.16	34.70	2.67	5.53	13.15	51.56	40.00	80.00
1	359	507	3	33	1507	1	767.50	19.35	4.10	12.28	14.78	52.33	51.00	88.00
1	360	508	3	33	1508	1	1106.78	35.40	3.17	8.36	11.70	49.00	35.00	77.00
1	361	509	3	33	1509	1	849.76	20.00	3.35	11.34	15.95	52.67	50.00	90.00
1	362	510	3	33	1510	1	1303.17	34.30	3.39	9.57	11.80	53.11	40.00	80.00
1	363	511	3	33	1511	1	682.38	17.10	3.57	9.73	13.78	52.00	51.00	88.00
2	364	1	4	34	21	1	1625.55	21.60	3.24	9.25	12.40	41.33	39.00	72.00
2	365	3	4	34	23	1	2400.88	30.50	4.02	11.58	11.80	50.00	34.00	72.00
2	366	5	4	34	25	1	2413.33	21.30	3.50	13.59	11.60	52.00	40.00	72.00
2	367	6	4	34	26	1	1307.50	36.40	2.47	10.50	13.60	46.33	32.00	87.00
2	368	11	4	34	211	1	1058.89	39.80	2.18	6.78	11.60	36.00	33.00	72.00
2	369	12	4	34	212	1	1811.50	20.40	4.00	11.50	14.60	55.33	46.00	87.00
2	370	13	4	34	213	1	120.50	18.90	3.50	2.00	14.90	51.67	61.00	92.00
2	371	15	4	34	215	1	1733.33	32.20	2.84	10.82	11.80	49.53	40.00	72.00
2	372	20	4	34	220	1	1674.44	36.60	3.54	6.32	12.00	53.00	40.00	80.00
2	373	22	4	34	222	1	1606.67	20.60	3.39	13.73	12.60	47.00	37.00	72.00
2	374	23	4	34	223	1	2790.00	32.60	4.09	11.16	13.80	58.86	47.00	87.00
2	375	24	4	35	224	1	1350.00	24.77	4.10	10.88	13.80	46.67	51.00	87.00
2	376	25	4	35	225	1	665.55	26.10	2.11	7.18	16.00	51.00	42.00	87.00
2	377	26	4	35	226	1	1527.78	21.70	3.00	9.22	13.60	59.00	51.00	80.00
2	378	27	4	35	227	1	1300.89	25.80	2.25	5.00	13.40	53.33	51.00	89.00
2	379	28	4	35	228	1	2073.33	20.30	4.30	14.81	16.40	51.00	52.00	87.00
2	380	29	4	35	229	1	968.89	19.30	4.09	10.64	12.20	50.50	51.00	87.00
2	381	30	4	35	230	1	2068.89	30.80	3.84	7.65	20.60	52.67	51.00	87.00
2	382	32	4	35	232	1	1133.33	19.00	3.44	8.78	14.80	51.00	51.00	87.00
2	383	33	4	35	233	1	2967.77	21.30	5.15	13.10	13.60	57.00	41.00	80.00

Year	Plot	Acessions	Replication	Block	Interaction	Observation /Plot	YDSD	SW	SDPD	PDPL	FPIH	PLHT	DF	DPM
2	384	34	4	35	234	1	440.00	21.00	2.27	9.80	20.80	55.33	46.00	92.00
2	385	35	4	35	235	1	2288.89	16.60	4.71	12.73	15.40	56.67	52.00	87.00
2	386	36	4	36	236	1	1234.44	17.60	3.83	13.07	11.40	40.67	43.00	80.00
2	387	37	4	36	237	1	1841.11	34.00	3.35	7.35	13.60	53.00	40.00	80.00
2	388	38	4	36	238	1	755.55	39.60	3.21	6.22	9.80	37.90	31.00	76.00
2	389	39	4	36	239	1	1740.00	34.90	3.13	11.00	12.20	43.33	32.00	80.00
2	390	40	4	36	240	1	1174.44	42.30	2.18	6.84	10.20	46.89	32.00	85.00
2	391	41	4	36	241	1	1614.44	41.50	2.23	10.28	11.80	39.33	31.00	76.00
2	392	42	4	36	242	1	1747.78	34.30	3.26	6.30	13.60	50.00	36.00	80.00
2	393	43	4	36	243	1	950.00	35.80	2.80	5.44	9.20	40.00	32.00	80.00
2	394	44	4	36	244	1	827.78	16.10	4.09	7.25	12.20	48.00	40.00	76.00
2	395	45	4	36	245	1	425.56	20.00	5.00	11.33	12.45	46.50	52.00	80.00
2	396	46	4	36	246	1	1908.89	18.10	3.76	9.13	13.40	45.66	39.00	80.00
2	397	47	4	37	247	1	286.00	14.99	2.13	4.66	11.50	48.50	51.00	80.00
2	398	49	4	37	249	1	2324.44	19.90	4.96	11.89	16.00	52.00	51.00	87.00
2	399	52	4	37	252	1	1732.22	25.20	3.56	11.00	14.60	40.67	39.00	80.00
2	400	56	4	37	256	1	2508.89	22.70	4.58	11.35	15.20	50.00	40.00	80.00
2	401	57	4	37	257	1	1296.67	21.70	3.89	9.88	14.60	49.00	52.00	87.00
2	402	58	4	37	258	1	281.11	23.20	2.13	3.31	22.90	53.33	48.00	92.00
2	403	60	4	37	260	1	2376.66	17.80	4.58	13.50	13.00	57.33	51.00	87.00
2	404	61	4	37	261	1	1822.22	35.70	3.57	7.56	12.00	53.00	32.00	72.00
2	405	62	4	37	262	1	1621.11	39.70	2.83	6.57	11.40	44.00	32.00	80.00
2	406	63	4	37	263	1	1100.00	18.00	4.56	5.33	12.50	50.77	48.00	80.00
2	407	64	4	37	264	1	1794.44	29.90	3.09	8.00	10.20	49.99	39.00	80.00
2	408	65	4	38	265	1	1926.66	34.10	3.34	10.60	13.60	51.33	39.00	80.00
2	409	66	4	38	266	1	1495.60	18.30	3.96	10.80	13.60	50.00	50.00	87.00
2	410	67	4	38	267	1	1824.44	34.00	3.28	8.32	13.00	44.00	31.00	80.00
2	411	69	4	38	269	1	2158.89	24.70	3.30	12.71	15.00	60.67	47.00	80.00
2	412	70	4	38	270	1	2006.66	29.40	3.37	8.71	13.20	56.00	40.00	80.00
2	413	71	4	38	271	1	1774.44	21.90	3.49	10.81	13.40	53.33	45.00	80.00
2	414	72	4	38	272	1	2715.55	17.60	5.86	12.30	15.40	53.00	47.00	80.00
2	415	73	4	38	273	1	1300.00	37.80	2.92	12.56	12.00	45.00	32.00	72.00

Year	Plot	Acessions	Replication	Block	Interaction	Observation /Plot	YDSD	SW	SDPD	PDPL	FPIH	PLHT	DF	DPM
2	416	74	4	38	274	1	845.55	18.70	3.99	7.47	12.80	40.00	41.00	80.00
2	417	75	4	38	275	1	1160.55	20.60	3.30	7.57	15.60	43.66	43.00	75.00
2	418	82	4	38	282	1	2411.11	38.10	3.72	10.73	14.40	55.67	39.00	80.00
2	419	83	4	39	283	1	1324.44	41.40	2.49	5.91	12.80	43.00	32.00	76.00
2	420	85	4	39	285	1	992.05	22.00	3.50	6.07	25.60	56.66	52.00	92.00
2	421	89	4	39	289	1	1378.89	41.80	2.93	8.54	12.20	41.67	34.00	80.00
2	422	91	4	39	291	1	1401.11	34.50	2.96	8.47	12.40	51.00	34.00	80.00
2	423	93	4	39	293	1	1377.78	36.00	2.82	6.05	12.60	46.33	32.00	80.00
2	424	94	4	39	294	1	1765.55	22.00	3.65	12.06	20.80	49.67	52.00	87.00
2	425	95	4	39	295	1	2394.67	16.00	4.66	8.29	12.00	49.67	52.00	87.00
2	426	97	4	39	297	1	1398.89	34.50	2.67	6.75	10.40	50.99	36.00	80.00
2	427	98	4	39	298	1	1541.11	27.60	3.58	6.59	12.40	43.00	39.00	72.00
2	428	99	4	39	299	1	2308.89	33.40	5.37	7.31	11.80	49.00	37.00	72.00
2	429	100	4	39	2100	1	1567.00	21.50	3.12	9.13	12.40	55.67	45.00	80.00
2	430	101	4	40	2101	1	1464.44	30.90	2.94	10.57	10.60	46.00	40.00	80.00
2	431	102	4	40	2102	1	2764.44	34.60	3.66	4.00	12.80	45.67	34.00	72.00
2	432	104	4	40	2104	1	1814.44	33.10	2.44	8.46	11.60	53.67	40.00	80.00
2	433	105	4	40	2105	1	1436.67	21.00	4.78	7.48	13.80	55.33	52.00	80.00
2	434	107	4	40	2107	1	1254.44	32.10	3.68	6.19	12.20	60.77	34.00	72.00
2	435	110	4	40	2110	1	1958.89	38.50	3.04	8.42	11.40	49.00	37.00	80.00
2	436	111	4	40	2111	1	2707.78	35.10	3.54	9.45	9.00	49.88	38.00	72.00
2	437	112	4	40	2112	1	1664.44	23.00	3.51	11.06	12.00	54.00	42.00	80.00
2	438	113	4	40	2113	1	1864.44	21.80	3.76	12.11	13.40	55.67	47.00	80.00
2	439	114	4	40	2114	1	1870.00	32.90	3.00	11.50	13.60	47.33	32.00	80.00
2	440	115	4	40	2115	1	1608.89	36.00	3.25	7.88	11.20	46.00	32.00	80.00
2	441	117	4	41	2117	1	2503.33	20.10	3.98	15.67	21.20	50.67	51.00	92.00
2	442	119	4	41	2119	1	1064.44	38.60	2.22	12.00	10.80	40.00	32.00	72.00
2	443	120	4	41	2120	1	1820.00	28.10	3.40	12.00	13.60	47.33	40.00	80.00
2	444	121	4	41	2121	1	711.11	19.10	3.80	4.76	12.20	54.00	39.00	80.00
2	445	128	4	41	2128	1	1440.00	34.90	2.44	7.10	13.80	52.67	40.00	80.00
2	446	138	4	41	2138	1	934.66	18.50	3.60	8.99	13.00	49.33	50.00	80.00
2	447	139	4	41	2139	1	1088.89	34.20	2.47	6.44	10.80	41.66	32.00	80.00

Year	Plot	Acessions	Replication	Block	Interaction	Observation /Plot	YDSD	SW	SDPD	PDPL	FPIH	PLHT	DF	DPM
2	448	144	4	41	2144	1	682.00	20.66	2.13	7.90	13.00	54.00	50.00	80.00
2	449	153	4	41	2153	1	1516.88	17.60	6.90	11.00	13.80	52.67	50.00	80.00
2	450	154	4	41	2154	1	753.33	36.50	2.76	9.71	15.60	46.00	32.00	75.00
2	451	156	4	41	2156	1	4282.22	24.50	4.44	18.50	15.20	50.67	49.00	87.00
2	452	161	4	42	2161	1	875.66	35.40	2.29	9.33	15.80	51.00	43.00	80.00
2	453	162	4	42	2162	1	621.11	37.90	2.08	3.95	14.40	50.01	32.00	80.00
2	454	163	4	42	2163	1	422.33	42.00	2.33	2.00	8.33	50.00	33.00	80.00
2	455	164	4	42	2164	1	1548.89	22.00	3.13	10.33	12.00	48.90	36.00	80.00
2	456	165	4	42	2165	1	1268.89	42.00	3.34	5.00	14.40	52.67	39.00	80.00
2	457	166	4	42	2166	1	121.11	32.05	1.62	3.50	12.40	46.67	33.00	87.00
2	458	167	4	42	2167	1	878.89	60.00	2.77	2.89	13.20	40.00	34.00	87.00
2	459	168	4	42	2168	1	2164.44	21.60	3.77	14.94	13.20	51.33	47.00	80.00
2	460	169	4	42	2169	1	1851.11	16.90	3.69	13.40	13.00	56.00	45.00	80.00
2	461	170	4	42	2170	1	1635.55	22.40	3.92	9.63	16.20	49.67	51.00	87.00
2	462	172	4	42	2172	1	1775.55	40.50	2.67	7.80	10.80	49.89	32.00	75.00
2	463	174	4	43	2174	1	1307.78	30.90	3.37	6.00	14.20	56.67	46.00	80.00
2	464	175	4	43	2175	1	386.67	28.50	3.91	16.00	12.10	54.00	40.00	80.00
2	465	188	4	43	2188	1	1263.33	34.60	3.94	4.78	11.00	45.66	37.00	75.00
2	466	191	4	43	2191	1	1026.67	35.00	3.05	6.50	14.60	50.33	42.00	80.00
2	467	194	4	43	2194	1	1602.22	34.80	2.69	8.22	12.40	58.89	40.00	75.00
2	468	197	4	43	2197	1	2177.78	37.80	3.48	7.38	12.40	52.33	40.00	80.00
2	469	199	4	43	2199	1	1444.50	24.30	2.72	9.00	12.80	49.00	46.00	72.00
2	470	200	4	43	2200	1	1707.22	15.80	8.09	3.25	12.60	51.00	51.00	80.00
2	471	201	4	43	2201	1	1255.55	23.40	3.52	10.63	14.60	49.78	52.00	89.00
2	472	205	4	43	2205	1	2330.00	20.40	4.10	16.06	13.20	49.90	52.00	87.00
2	473	500	4	43	2500	1	1440.00	20.80	3.00	10.14	12.20	58.67	52.00	80.00
2	474	501	4	44	2501	1	1385.55	25.30	3.37	8.10	15.00	50.67	51.00	87.00
2	475	502	4	44	2502	1	2790.00	20.30	5.73	9.83	14.20	55.67	49.00	87.00
2	476	503	4	44	2503	1	2936.66	25.80	4.56	14.25	14.40	53.33	43.00	80.00
2	477	504	4	44	2504	1	2490.99	22.80	3.74	11.90	13.00	57.00	47.00	87.00
2	478	505	4	44	2505	1	718.89	19.00	3.21	5.59	16.40	64.00	52.00	80.00
2	479	506	4	44	2506	1	1752.22	39.60	2.69	8.11	13.80	52.67	40.00	80.00

Year	Plot	Acessions	Replication	Block	Interaction	Observation /Plot	YDSD	SW	SDPD	PDPL	FPIH	PLHT	DF	DPM
2	480	507	4	44	2507	1	1109.56	17.10	3.99	9.64	13.00	52.00	51.00	80.00
2	481	508	4	44	2508	1	1576.67	31.90	3.07	6.08	12.60	49.77	36.00	72.00
2	482	509	4	44	2509	1	1822.40	18.90	2.50	11.90	16.20	50.66	50.00	92.00
2	483	510	4	44	2510	1	2473.33	32.50	3.80	9.15	11.80	53.22	37.00	80.00
2	484	511	4	44	2511	1	1292.35	15.00	3.77	9.90	15.20	50.00	52.00	87.00
2	485	1	5	45	21	1	1816.66	23.70	3.59	10.50	13.60	40.33	39.00	80.00
2	486	3	5	45	23	1	2670.00	30.60	4.91	9.41	12.60	51.00	34.00	80.00
2	487	5	5	45	25	1	1637.78	21.50	4.31	8.75	11.60	48.67	41.00	80.00
2	488	6	5	45	26	1	1342.22	37.40	2.39	13.09	15.60	43.67	32.00	80.00
2	489	11	5	45	211	1	1474.44	40.00	2.84	7.59	12.20	36.00	32.00	80.00
2	490	12	5	45	212	1	1831.11	23.20	4.20	11.06	14.40	56.33	46.00	80.00
2	491	13	5	45	213	1	173.33	20.26	2.57	2.00	16.40	55.00	61.00	92.00
2	492	15	5	45	215	1	2814.44	33.90	3.75	12.18	12.80	52.33	40.00	80.00
2	493	20	5	45	220	1	1332.22	26.70	3.33	7.67	13.80	54.00	39.00	80.00
2	494	22	5	45	222	1	2097.78	24.50	3.35	23.00	11.20	45.33	38.00	80.00
2	495	23	5	45	223	1	2926.66	31.10	4.15	9.83	14.20	59.00	52.00	87.00
2	496	24	5	46	224	1	4215.55	25.80	6.60	15.87	12.60	48.67	51.00	87.00
2	497	25	5	46	225	1	1432.22	25.10	3.46	10.94	18.00	51.67	42.00	87.00
2	498	26	5	46	226	1	2071.11	26.50	4.17	10.63	14.00	62.00	47.00	80.00
2	499	27	5	46	227	1	1500.00	32.70	2.69	9.83	18.60	53.67	51.00	92.00
2	500	28	5	46	228	1	2788.89	24.10	5.93	16.06	16.00	50.33	52.00	87.00
2	501	29	5	46	229	1	1013.33	22.10	3.80	10.43	13.60	48.33	52.00	87.00
2	502	30	5	46	230	1	2706.66	31.80	4.12	11.44	14.40	51.33	48.00	87.00
2	503	32	5	46	232	1	2233.33	19.50	5.24	11.65	12.80	49.00	51.00	87.00
2	504	33	5	46	233	1	1862.22	22.80	4.52	16.08	14.60	58.33	41.00	80.00
2	505	34	5	46	234	1	886.43	14.30	2.60	4.17	19.80	58.67	51.00	92.00
2	506	35	5	46	235	1	2678.89	17.90	5.99	12.37	13.60	50.67	51.00	89.00
2	507	36	5	47	236	1	2074.44	18.90	3.79	14.15	13.80	40.00	43.00	80.00
2	508	37	5	47	237	1	1692.22	31.40	3.59	8.63	12.33	54.67	39.00	80.00
2	509	38	5	47	238	1	1312.22	40.80	3.24	10.56	11.40	37.00	32.00	80.00
2	510	39	5	47	239	1	2203.33	37.30	3.20	8.50	12.40	40.78	32.00	80.00
2	511	40	5	47	240	1	1581.11	39.80	3.07	7.22	12.60	46.00	32.00	80.00

Year	Plot	Acessions	Replication	Block	Interaction	Observation /Plot	YDSD	SW	SDPD	PDPL	FPIH	PLHT	DF	DPM
2	512	41	5	47	241	1	2774.44	41.50	2.87	9.38	12.80	39.66	32.00	80.00
2	513	42	5	47	242	1	1651.11	36.30	3.40	6.50	13.80	54.00	39.00	80.00
2	514	43	5	47	243	1	1768.89	30.80	3.39	7.38	13.00	38.00	32.00	80.00
2	515	44	5	47	244	1	1811.11	18.20	5.27	13.33	14.00	45.67	40.00	80.00
2	516	45	5	47	245	1	746.67	20.60	4.76	10.71	11.90	47.99	52.00	87.00
2	517	46	5	47	246	1	3373.33	17.60	4.51	20.55	14.20	46.00	40.00	80.00
2	518	47	5	48	247	1	550.00	16.70	3.26	5.14	13.33	49.67	51.00	80.00
2	519	49	5	48	249	1	2260.00	23.60	4.34	11.20	18.20	52.67	51.00	87.00
2	520	52	5	48	252	1	2438.89	23.40	5.07	10.37	12.00	44.00	39.00	80.00
2	521	56	5	48	256	1	2482.88	15.60	4.26	8.89	11.40	50.67	40.00	87.00
2	522	57	5	48	257	1	1957.78	24.40	3.28	9.50	13.40	51.67	52.00	87.00
2	523	58	5	48	258	1	346.67	25.10	1.90	4.44	21.40	56.00	45.00	92.00
2	524	60	5	48	260	1	1545.55	18.00	3.68	12.50	13.40	52.33	47.00	87.00
2	525	61	5	48	261	1	2711.11	31.40	4.18	9.80	14.20	52.00	32.00	80.00
2	526	62	5	48	262	1	1427.78	38.40	2.84	7.11	12.40	45.67	32.00	80.00
2	527	63	5	48	263	1	1775.55	18.90	4.14	12.18	9.80	51.00	48.00	80.00
2	528	64	5	48	264	1	1794.44	31.20	2.55	9.65	12.80	48.33	39.00	80.00
2	529	65	5	49	265	1	2345.55	37.60	3.36	9.68	15.00	53.67	39.00	80.00
2	530	66	5	49	266	1	2488.89	20.00	5.10	11.82	12.80	50.67	52.00	87.00
2	531	67	5	49	267	1	1767.78	31.80	2.52	9.67	10.60	43.33	32.00	80.00
2	532	69	5	49	269	1	2752.22	23.70	6.55	8.00	14.80	58.66	52.00	87.00
2	533	70	5	49	270	1	2583.33	29.30	4.13	9.05	13.60	50.00	38.00	80.00
2	534	71	5	49	271	1	2052.22	21.00	4.06	12.48	11.60	54.67	45.00	80.00
2	535	72	5	49	272	1	2967.77	16.30	5.64	13.39	12.80	59.67	51.00	80.00
2	536	73	5	49	273	1	2023.33	37.70	3.36	6.48	14.00	43.00	32.00	80.00
2	537	74	5	49	274	1	1060.00	18.40	2.76	8.18	10.40	42.67	41.00	80.00
2	538	75	5	49	275	1	1725.55	24.60	3.60	10.47	12.80	43.67	43.00	80.00
2	539	82	5	49	282	1	4162.22	41.50	3.39	11.25	12.40	55.00	39.00	80.00
2	540	83	5	50	283	1	2597.78	43.00	2.96	6.89	15.40	44.33	32.00	80.00
2	541	85	5	50	285	1	1205.55	23.70	4.05	8.25	19.40	54.33	51.00	92.00
2	542	89	5	50	289	1	1576.88	38.60	2.61	9.64	11.00	40.00	34.00	80.00
2	543	91	5	50	291	1	1933.33	35.30	3.27	13.33	11.60	50.00	34.00	80.00

Year	Plot	Acessions	Replication	Block	Interaction	Observation /Plot	YDSD	SW	SDPD	PDPL	FPIH	PLHT	DF	DPM
2	544	93	5	50	293	1	2211.11	38.70	3.36	12.00	10.80	45.33	32.00	80.00
2	545	94	5	50	294	1	1813.33	16.80	5.55	13.86	15.20	51.00	52.00	87.00
2	546	95	5	50	295	1	4945.55	20.50	5.83	18.81	14.00	52.33	51.00	89.00
2	547	97	5	50	297	1	1997.78	36.90	3.05	6.90	13.20	48.33	38.00	80.00
2	548	98	5	50	298	1	2982.22	31.90	3.48	13.32	13.40	42.00	38.00	72.00
2	549	99	5	50	299	1	1580.00	33.10	3.19	6.76	11.20	47.67	38.00	72.00
2	550	100	5	50	2100	1	2851.11	25.70	3.75	15.05	12.80	52.00	51.00	80.00
2	551	101	5	51	2101	1	1855.55	31.40	3.96	6.71	12.00	48.67	38.00	80.00
2	552	102	5	51	2102	1	2234.90	32.10	3.90	4.50	13.60	49.33	34.00	72.00
2	553	104	5	51	2104	1	1603.33	34.40	3.18	6.85	14.60	59.67	39.00	80.00
2	554	105	5	51	2105	1	2393.33	19.10	4.63	12.05	13.60	58.00	51.00	80.00
2	555	107	5	51	2107	1	1833.33	33.60	3.72	8.24	11.60	63.33	34.00	72.00
2	556	110	5	51	2110	1	2291.11	33.80	4.00	8.50	13.40	50.00	37.00	80.00
2	557	111	5	51	2111	1	1926.66	33.60	3.57	8.33	14.40	48.00	38.00	72.00
2	558	112	5	51	2112	1	1862.34	21.70	2.27	9.19	12.00	51.00	42.00	80.00
2	559	113	5	51	2113	1	3782.22	21.00	4.50	17.23	12.20	58.67	51.00	80.00
2	560	114	5	51	2114	1	2174.44	35.20	3.35	9.00	13.00	50.67	32.00	80.00
2	561	115	5	51	2115	1	2201.11	42.60	3.20	9.00	13.00	47.00	32.00	80.00
2	562	117	5	52	2117	1	1443.33	21.00	4.35	12.27	20.40	51.33	51.00	92.00
2	563	119	5	52	2119	1	1784.44	42.70	3.17	12.70	11.80	40.00	32.00	80.00
2	564	120	5	52	2120	1	2543.33	28.40	3.57	13.12	12.60	48.67	40.00	80.00
2	565	121	5	52	2121	1	810.00	21.20	2.64	9.38	13.20	52.33	39.00	80.00
2	566	128	5	52	2128	1	2420.00	31.50	4.00	9.10	10.40	51.00	39.00	80.00
2	567	138	5	52	2138	1	1475.55	22.00	4.14	10.53	18.40	54.67	51.00	87.00
2	568	139	5	52	2139	1	923.33	36.60	2.85	5.00	13.40	40.67	32.00	80.00
2	569	144	5	52	2144	1	922.50	20.10	3.54	5.61	14.40	55.00	50.00	80.00
2	570	153	5	52	2153	1	2898.89	18.70	7.40	11.05	16.60	51.33	51.00	87.00
2	571	154	5	52	2154	1	1090.06	34.70	3.17	10.00	14.80	46.67	32.00	75.00
2	572	156	5	52	2156	1	3701.11	22.50	5.90	16.38	12.80	49.00	51.00	87.00
2	573	161	5	53	2161	1	1103.33	39.20	2.72	6.06	13.00	51.33	42.00	80.00
2	574	162	5	53	2162	1	1057.78	37.20	2.10	4.78	16.00	49.00	32.00	80.00
2	575	163	5	53	2163	1	572.22	45.20	1.65	9.63	13.00	51.00	32.00	80.00

Year	Plot	Acessions	Replication	Block	Interaction	Observation /Plot	YDSD	SW	SDPD	PDPL	FPIH	PLHT	DF	DPM
2	576	164	5	53	2164	1	1774.44	22.40	3.52	12.21	15.00	47.67	38.00	80.00
2	577	165	5	53	2165	1	751.11	36.40	2.72	4.35	12.20	52.33	39.00	80.00
2	578	166	5	53	2166	1	255.56	36.51	1.62	7.80	12.60	44.33	32.00	87.00
2	579	167	5	53	2167	1	575.55	49.60	2.20	2.27	15.60	40.00	32.00	87.00
2	580	168	5	53	2168	1	1414.44	18.50	2.69	14.00	14.20	54.33	47.00	80.00
2	581	169	5	53	2169	1	3400.00	19.90	4.06	16.50	12.60	59.33	46.00	80.00
2	582	170	5	53	2170	1	2575.55	18.60	4.63	12.64	14.20	51.00	50.00	87.00
2	583	172	5	53	2172	1	2251.11	39.70	3.14	9.95	14.20	48.33	32.00	80.00
2	584	174	5	54	2174	1	1412.50	25.30	3.36	6.35	15.60	53.67	45.00	80.00
2	585	175	5	54	2175	1	814.44	31.60	4.47	14.25	11.60	54.00	40.00	80.00
2	586	188	5	54	2188	1	2681.11	34.30	4.31	10.12	13.20	47.90	37.00	80.00
2	587	191	5	54	2191	1	1080.00	32.40	2.20	6.25	13.40	50.67	39.00	80.00
2	588	194	5	54	2194	1	2826.66	45.00	3.89	6.67	13.60	58.90	39.00	80.00
2	589	197	5	54	2197	1	2285.55	37.80	3.77	8.05	12.60	54.33	40.00	80.00
2	590	199	5	54	2199	1	2303.33	24.10	4.16	12.32	13.40	47.67	47.00	75.00
2	591	200	5	54	2200	1	3621.11	19.30	4.59	19.40	13.40	50.67	51.00	87.00
2	592	201	5	54	2201	1	1437.78	23.60	2.96	15.05	18.80	49.67	52.00	87.00
2	593	205	5	54	2205	1	2157.78	18.50	4.37	20.29	13.00	49.00	52.00	87.00
2	594	500	5	54	2500	1	1600.90	19.89	3.30	8.90	12.00	56.99	52.00	80.00
2	595	501	5	55	2501	1	1245.55	23.30	2.97	9.16	17.20	53.67	51.00	87.00
2	596	502	5	55	2502	1	3900.00	22.70	6.82	10.73	12.00	52.90	51.00	87.00
2	597	503	5	55	2503	1	2894.44	24.00	4.43	14.22	14.80	56.00	43.00	80.00
2	598	504	5	55	2504	1	4290.00	26.90	5.89	12.32	16.00	57.33	51.00	87.00
2	599	505	5	55	2505	1	872.22	18.60	3.48	9.06	18.60	58.00	52.00	80.00
2	600	506	5	55	2506	1	2348.89	34.40	3.58	7.83	13.60	52.67	40.00	80.00
2	601	507	5	55	2507	1	2876.66	19.90	4.91	16.72	14.00	50.33	51.00	87.00
2	602	508	5	55	2508	1	1866.66	32.90	4.16	6.50	12.40	47.67	34.00	80.00
2	603	509	5	55	2509	1	2241.11	23.80	3.60	13.00	16.80	52.33	50.00	92.00
2	604	510	5	55	2510	1	2685.55	33.90	3.97	9.89	11.00	51.00	39.00	80.00
2	605	511	5	55	2511	1	1383.33	15.70	4.28	11.60	11.60	50.00	52.00	87.00
2	606	1	6	56	21	1	1434.44	21.80	3.22	9.09	11.60	40.33	39.00	75.00
2	607	3	6	56	23	1	2481.11	34.20	3.14	12.65	11.20	53.00	34.00	75.00

Year	Plot	Acessions	Replication	Block	Interaction	Observation /Plot	YDSD	SW	SDPD	PDPL	FPIH	PLHT	DF	DPM
2	608	5	6	56	25	1	2020.89	19.90	3.74	9.56	11.60	48.00	42.00	80.00
2	609	6	6	56	26	1	1273.33	40.10	2.55	7.29	11.60	44.55	32.00	80.00
2	610	11	6	56	211	1	1260.00	41.30	2.50	7.20	12.50	35.33	32.00	80.00
2	611	12	6	56	212	1	1792.22	19.10	4.28	12.89	15.00	53.33	46.00	80.00
2	612	13	6	56	213	1	150.00	19.50	3.20	2.50	15.10	52.00	61.00	92.00
2	613	15	6	56	215	1	2270.00	36.20	2.53	11.48	13.00	47.33	40.00	80.00
2	614	20	6	56	220	1	1300.00	29.80	3.26	6.63	12.40	54.00	39.00	80.00
2	615	22	6	56	222	1	2816.66	26.30	3.97	10.87	10.30	43.87	38.00	75.00
2	616	23	6	56	223	1	2406.66	31.70	4.03	9.76	14.80	57.67	49.00	87.00
2	617	24	6	57	224	1	2782.50	23.10	5.50	13.83	13.60	45.67	51.00	80.00
2	618	25	6	57	225	1	583.33	21.30	1.86	7.24	15.40	50.67	42.00	80.00
2	619	26	6	57	226	1	1482.22	22.90	4.00	10.50	14.00	57.00	49.00	80.00
2	620	27	6	57	227	1	876.67	30.10	2.68	5.55	14.60	51.33	51.00	90.00
2	621	28	6	57	228	1	1510.00	19.90	4.50	15.90	16.00	50.00	52.00	87.00
2	622	29	6	57	229	1	1373.33	22.30	4.40	10.57	12.80	52.00	52.00	87.00
2	623	30	6	57	230	1	2387.00	30.90	4.00	10.90	17.60	54.33	49.00	92.00
2	624	32	6	57	232	1	2151.11	18.90	4.28	13.00	15.20	50.00	51.00	87.00
2	625	33	6	57	233	1	1661.89	19.90	3.84	13.08	16.80	55.33	40.00	80.00
2	626	34	6	57	234	1	550.69	16.00	2.50	4.90	20.00	59.00	49.00	92.00
2	627	35	6	57	235	1	2365.55	18.70	6.04	13.44	15.40	53.67	52.00	87.00
2	628	36	6	58	236	1	1087.55	17.10	4.69	10.77	11.60	40.44	43.00	80.00
2	629	37	6	58	237	1	1444.44	36.30	3.12	6.94	12.60	52.33	38.00	80.00
2	630	38	6	58	238	1	1381.11	40.00	3.17	14.00	10.80	38.90	32.00	80.00
2	631	39	6	58	239	1	1647.78	34.30	3.00	12.50	13.40	42.55	32.00	80.00
2	632	40	6	58	240	1	1038.89	38.50	3.60	7.88	12.00	46.00	32.00	92.00
2	633	41	6	58	241	1	2270.00	38.40	2.94	11.29	13.80	40.00	32.00	80.00
2	634	42	6	58	242	1	1457.78	35.90	3.22	5.81	13.40	50.00	38.00	75.00
2	635	43	6	58	243	1	1203.33	34.40	3.00	6.40	11.56	39.00	32.00	80.00
2	636	44	6	58	244	1	812.22	19.40	3.44	8.93	12.20	47.00	40.00	80.00
2	637	45	6	58	245	1	584.44	21.90	5.10	10.80	13.10	45.33	52.00	80.00
2	638	46	6	58	246	1	1940.34	18.90	5.00	13.43	14.55	48.67	40.00	80.00
2	639	47	6	59	247	1	465.90	18.90	2.45	4.10	12.55	49.00	51.00	75.00

Year	Plot	Acessions	Replication	Block	Interaction	Observation /Plot	YDSD	SW	SDPD	PDPL	FPIH	PLHT	DF	DPM
2	640	49	6	59	249	1	2014.00	19.30	4.56	11.10	17.00	51.00	51.00	87.00
2	641	52	6	59	252	1	2047.78	25.40	4.48	10.88	12.60	41.67	39.00	80.00
2	642	56	6	59	256	1	1312.22	20.90	4.04	8.20	14.60	53.67	40.00	87.00
2	643	57	6	59	257	1	1701.44	22.80	3.50	9.66	12.60	50.00	52.00	80.00
2	644	58	6	59	258	1	298.89	22.90	1.67	4.56	25.33	56.33	46.00	92.00
2	645	60	6	59	260	1	1318.89	15.60	2.16	10.36	14.20	54.33	49.00	87.00
2	646	61	6	59	261	1	1795.55	30.10	3.50	12.69	14.20	50.00	32.00	75.00
2	647	62	6	59	262	1	1446.67	40.10	2.71	6.80	12.60	44.67	32.00	80.00
2	648	63	6	59	263	1	1493.33	17.50	3.68	6.08	15.20	50.67	48.00	80.00
2	649	64	6	59	264	1	1698.89	30.80	3.79	6.43	14.40	48.33	39.00	80.00
2	650	65	6	60	265	1	1822.22	35.20	2.71	13.07	12.20	53.67	39.00	80.00
2	651	66	6	60	266	1	1511.88	19.78	3.70	10.18	11.80	49.67	52.00	87.00
2	652	67	6	60	267	1	1270.00	30.40	2.38	10.44	10.80	44.00	32.00	80.00
2	653	69	6	60	269	1	2234.88	22.55	4.50	10.77	14.00	55.66	49.00	80.00
2	654	70	6	60	270	1	1394.44	29.60	3.60	7.88	13.30	52.33	40.00	80.00
2	655	71	6	60	271	1	1794.44	22.20	3.27	11.00	10.20	55.33	45.00	80.00
2	656	72	6	60	272	1	2841.33	15.90	5.10	11.40	14.10	56.00	49.00	80.00
2	657	73	6	60	273	1	1940.00	37.50	3.00	8.76	12.00	44.33	33.00	80.00
2	658	74	6	60	274	1	827.78	19.20	3.43	10.31	12.60	40.00	41.00	80.00
2	659	75	6	60	275	1	1594.44	24.90	4.34	8.59	9.40	46.33	43.00	75.00
2	660	82	6	60	282	1	2408.89	37.80	3.44	10.75	15.60	55.33	39.00	80.00
2	661	83	6	61	283	1	1550.55	38.70	2.86	5.69	12.80	43.33	32.00	80.00
2	662	85	6	61	285	1	1182.44	23.10	3.80	2.47	22.00	55.33	51.00	92.00
2	663	89	6	61	289	1	1211.90	39.00	2.50	7.80	12.00	41.00	34.00	80.00
2	664	91	6	61	291	1	1497.78	36.30	3.00	11.90	12.10	50.00	34.00	80.00
2	665	93	6	61	293	1	1245.55	40.10	2.65	4.63	13.60	45.00	32.00	80.00
2	666	94	6	61	294	1	1262.11	18.60	4.50	11.70	13.40	48.77	52.00	87.00
2	667	95	6	61	295	1	3533.99	17.80	4.47	10.37	12.20	51.67	51.00	87.00
2	668	97	6	61	297	1	1446.67	38.20	2.90	5.50	14.40	50.67	38.00	80.00
2	669	98	6	61	298	1	1981.11	31.90	3.50	11.90	10.20	42.33	39.00	75.00
2	670	99	6	61	299	1	1462.22	32.70	2.85	9.38	10.00	48.33	39.00	75.00
2	671	100	6	61	2100	1	1876.40	19.10	3.50	13.80	12.10	53.33	48.00	80.00

Year	Plot	Acessions	Replication	Block	Interaction	Observation /Plot	YDSD	SW	SDPD	PDPL	FPIH	PLHT	DF	DPM
2	672	101	6	62	2101	1	1952.22	31.90	3.67	8.00	12.40	48.33	40.00	80.00
2	673	102	6	62	2102	1	1911.60	30.60	3.45	4.75	9.20	47.33	34.00	75.00
2	674	104	6	62	2104	1	2187.78	37.00	3.20	9.72	13.00	56.44	40.00	80.00
2	675	105	6	62	2105	1	1551.99	16.30	4.17	6.86	12.40	56.77	52.00	80.00
2	676	107	6	62	2107	1	1444.44	30.60	3.57	8.33	9.60	59.00	34.00	75.00
2	677	110	6	62	2110	1	1366.67	34.60	2.94	9.07	12.40	47.67	37.00	80.00
2	678	111	6	62	2111	1	2093.33	36.20	3.69	8.27	9.60	49.67	38.00	75.00
2	679	112	6	62	2112	1	2061.11	23.10	3.65	11.60	12.60	53.00	42.00	80.00
2	680	113	6	62	2113	1	2501.11	20.80	5.59	10.45	13.60	56.87	49.00	80.00
2	681	114	6	62	2114	1	2022.50	33.40	3.17	7.00	14.00	48.66	32.00	80.00
2	682	115	6	62	2115	1	1904.67	34.50	3.40	8.70	12.00	48.33	32.00	87.00
2	683	117	6	63	2117	1	1973.00	18.60	3.43	13.89	22.33	50.33	51.00	92.00
2	684	119	6	63	2119	1	1424.00	39.40	2.08	12.30	10.00	40.33	32.00	80.00
2	685	120	6	63	2120	1	1987.78	28.60	4.82	11.31	10.20	46.00	40.00	75.00
2	686	121	6	63	2121	1	562.22	18.20	2.69	6.77	12.50	50.00	39.00	80.00
2	687	128	6	63	2128	1	1872.22	34.60	3.20	8.58	13.60	51.33	40.00	80.00
2	688	138	6	63	2138	1	1845.55	20.80	4.80	8.80	12.40	50.67	51.00	87.00
2	689	139	6	63	2139	1	1246.67	38.00	2.50	5.90	11.90	41.00	32.00	80.00
2	690	144	6	63	2144	1	1163.33	19.50	2.90	9.74	12.60	56.00	50.00	80.00
2	691	153	6	63	2153	1	1902.22	19.10	4.94	11.41	14.00	54.67	51.00	80.00
2	692	154	6	63	2154	1	890.66	32.90	2.60	13.33	14.67	46.66	32.00	75.00
2	693	156	6	63	2156	1	3991.55	21.60	5.33	13.44	12.60	50.00	49.00	87.00
2	694	161	6	64	2161	1	1574.44	36.40	2.51	11.57	12.20	52.00	42.00	80.00
2	695	162	6	64	2162	1	951.11	37.66	2.00	4.90	13.00	48.33	32.00	80.00
2	696	163	6	64	2163	1	502.22	44.30	1.46	2.60	12.00	51.33	33.00	80.00
2	697	164	6	64	2164	1	1508.99	21.50	3.70	11.90	13.00	46.00	36.00	80.00
2	698	165	6	64	2165	1	778.89	38.10	2.90	4.60	12.66	53.44	39.00	80.00
2	699	166	6	64	2166	1	222.45	36.25	1.60	3.90	12.00	45.90	32.00	87.00
2	700	167	6	64	2167	1	434.90	55.90	1.38	1.05	15.40	40.66	33.00	87.00
2	701	168	6	64	2168	1	1223.90	23.20	2.40	14.00	15.40	50.00	47.00	80.00
2	702	169	6	64	2169	1	2112.22	20.30	3.46	10.50	12.40	58.00	46.00	80.00
2	703	170	6	64	2170	1	1223.33	20.89	4.60	10.66	15.30	51.90	51.00	87.00

Year	Plot	Acessions	Replication	Block	Interaction	Observation /Plot	YDSD	SW	SDPD	PDPL	FPIH	PLHT	DF	DPM
2	704	172	6	64	2172	1	1908.89	37.30	2.11	7.67	12.90	48.66	32.00	75.00
2	705	174	6	65	2174	1	1518.89	30.90	3.21	8.56	14.10	52.50	46.00	80.00
2	706	175	6	65	2175	1	418.89	28.90	2.83	27.00	12.00	54.70	40.00	80.00
2	707	188	6	65	2188	1	3105.55	37.20	4.21	8.04	13.80	46.66	37.00	75.00
2	708	191	6	65	2191	1	1396.67	35.40	3.25	6.72	14.20	51.00	40.00	80.00
2	709	194	6	65	2194	1	2055.55	35.00	3.99	8.44	13.60	60.00	40.00	75.00
2	710	197	6	65	2197	1	1800.00	35.00	3.34	5.11	10.40	54.67	40.00	80.00
2	711	199	6	65	2199	1	1586.67	24.00	5.81	9.08	11.80	46.66	47.00	75.00
2	712	200	6	65	2200	1	2286.12	19.00	3.49	8.59	16.00	52.00	51.00	87.00
2	713	201	6	65	2201	1	1266.67	23.90	3.10	13.90	13.40	52.67	52.00	87.00
2	714	205	6	65	2205	1	1396.67	17.90	3.98	9.00	12.20	49.67	52.00	87.00
2	715	500	6	65	2500	1	1069.77	18.30	3.67	6.24	12.20	54.60	52.00	80.00
2	716	501	6	66	2501	1	935.55	23.60	2.27	17.10	13.40	50.67	51.00	87.00
2	717	502	6	66	2502	1	2756.66	22.70	5.87	10.90	12.90	53.00	49.00	87.00
2	718	503	6	66	2503	1	1955.90	23.70	3.94	12.23	13.20	53.90	43.00	75.00
2	719	504	6	66	2504	1	2603.33	23.90	5.32	10.50	13.20	56.00	49.00	87.00
2	720	505	6	66	2505	1	845.33	15.00	3.52	5.24	22.60	60.00	52.00	80.00
2	721	506	6	66	2506	1	1588.70	34.40	3.50	7.90	13.10	53.11	40.00	80.00
2	722	507	6	66	2507	1	1790.88	21.50	4.30	14.90	15.00	52.33	51.00	87.00
2	723	508	6	66	2508	1	1884.44	33.40	3.08	8.65	11.00	48.00	34.00	80.00
2	724	509	6	66	2509	1	1930.77	21.00	3.50	10.88	16.90	55.33	50.00	92.00
2	725	510	6	66	2510	1	2385.55	31.20	3.48	12.88	13.80	53.88	39.00	80.00
2	726	511	6	66	2511	1	1547.78	17.30	4.70	10.05	13.00	49.67	52.00	87.00
3	727	1	7	67	31	1	686.67	23.00	2.02	9.14	14.60	38.00	39.00	73.00
3	728	3	7	67	33	1	1207.78	33.50	2.30	9.86	12.40	50.67	34.00	73.00
3	729	5	7	67	35	1	717.78	21.40	2.57	14.78	13.20	42.67	40.00	73.00
3	730	6	7	67	36	1	1238.89	41.80	2.05	7.44	10.20	43.33	32.00	73.00
3	731	11	7	67	311	1	937.78	42.00	2.26	8.27	12.80	39.00	33.00	73.00
3	732	12	7	67	312	1	1508.89	23.10	3.78	9.05	14.40	60.00	45.00	88.00
3	733	13	7	67	313	1	144.44	16.60	3.73	3.67	16.00	50.67	60.00	80.00
3	734	15	7	67	315	1	835.55	31.00	3.57	7.64	15.00	54.67	40.00	73.00
3	735	20	7	67	320	1	1130.00	41.00	3.51	6.25	11.60	51.67	40.00	80.00

Year	Plot	Acessions	Replication	Block	Interaction	Observation /Plot	YDSD	SW	SDPD	PDPL	FPIH	PLHT	DF	DPM
3	736	22	7	67	322	1	1275.55	27.00	3.37	9.29	12.00	53.67	38.00	73.00
3	737	23	7	67	323	1	3353.33	32.50	3.06	20.00	14.80	64.33	49.00	88.00
3	738	24	7	68	324	1	1544.44	27.00	3.43	13.21	13.00	51.00	50.00	88.00
3	739	25	7	68	325	1	1264.44	23.20	2.94	12.35	16.00	52.00	40.00	88.00
3	740	26	7	68	326	1	1086.67	24.60	2.67	13.92	12.80	50.00	50.00	80.00
3	741	27	7	68	327	1	578.89	23.30	3.33	9.13	13.60	50.00	50.00	88.00
3	742	28	7	68	328	1	1364.44	25.00	3.85	11.29	11.00	50.00	50.00	88.00
3	743	29	7	68	329	1	948.89	22.50	4.43	12.00	12.20	50.00	50.00	88.00
3	744	30	7	68	330	1	1031.11	31.20	2.21	15.10	15.00	50.67	50.00	88.00
3	745	32	7	68	332	1	1735.55	20.90	3.18	12.88	12.50	49.33	50.00	88.00
3	746	33	7	68	333	1	1255.55	24.80	3.73	10.77	14.40	52.33	40.00	88.00
3	747	34	7	68	334	1	1124.44	21.60	2.70	15.00	15.40	53.33	48.00	88.00
3	748	35	7	68	335	1	376.67	16.20	2.06	17.50	15.60	60.00	50.00	88.00
3	749	36	7	69	336	1	671.11	21.40	3.11	8.36	11.20	45.33	45.00	80.00
3	750	37	7	69	337	1	2340.00	36.60	2.61	10.90	13.60	54.33	40.00	73.00
3	751	38	7	69	338	1	625.55	41.00	2.42	4.62	9.40	38.67	30.00	73.00
3	752	39	7	69	339	1	988.89	38.40	2.99	11.83	13.40	39.67	32.00	73.00
3	753	40	7	69	340	1	2135.55	46.60	3.03	8.29	13.00	47.33	35.00	73.00
3	754	41	7	69	341	1	1502.22	38.60	2.57	6.90	11.40	42.00	35.00	73.00
3	755	42	7	69	342	1	1677.78	40.60	3.25	5.43	13.20	41.33	40.00	73.00
3	756	43	7	69	343	1	1373.33	33.60	2.33	4.78	10.20	40.67	38.00	73.00
3	757	44	7	69	344	1	695.55	20.40	3.49	7.69	11.20	46.67	40.00	80.00
3	758	45	7	69	345	1	797.78	17.60	3.56	7.65	17.00	48.67	50.00	80.00
3	759	46	7	69	346	1	1302.22	21.80	3.24	13.71	11.40	42.67	40.00	73.00
3	760	47	7	70	347	1	200.56	12.80	3.65	3.50	10.00	42.00	50.00	80.00
3	761	49	7	70	349	1	562.22	21.80	2.38	7.82	16.00	50.00	50.00	88.00
3	762	52	7	70	352	1	1040.00	27.60	2.94	12.60	14.00	46.33	40.00	80.00
3	763	56	7	70	356	1	1586.67	27.00	3.04	20.20	13.00	46.67	40.00	80.00
3	764	57	7	70	357	1	813.33	24.40	2.26	9.93	13.20	49.00	50.00	80.00
3	765	58	7	70	358	1	451.11	25.40	1.76	7.50	16.40	48.33	50.00	88.00
3	766	60	7	70	360	1	502.22	17.40	2.13	11.92	15.60	50.67	50.00	88.00
3	767	61	7	70	361	1	1257.78	34.40	2.47	6.57	13.20	51.67	35.00	73.00

Year	Plot	Acessions	Replication	Block	Interaction	Observation /Plot	YDSD	SW	SDPD	PDPL	FPIH	PLHT	DF	DPM
3	768	62	7	70	362	1	851.11	39.80	2.65	5.75	14.00	49.67	30.00	73.00
3	769	63	7	70	363	1	766.67	20.66	3.52	10.23	12.20	50.22	50.00	80.00
3	770	64	7	70	364	1	1464.44	36.80	3.81	5.16	11.40	50.00	40.00	73.00
3	771	65	7	71	365	1	1837.78	41.00	3.04	7.14	14.80	44.67	40.00	80.00
3	772	66	7	71	366	1	864.44	19.60	3.35	14.70	13.80	46.67	50.00	88.00
3	773	67	7	71	367	1	696.67	36.40	2.43	5.69	12.20	35.00	30.00	73.00
3	774	69	7	71	369	1	607.78	22.30	3.79	13.20	11.20	47.67	50.00	88.00
3	775	70	7	71	370	1	1933.33	36.40	3.29	11.29	14.80	42.00	40.00	80.00
3	776	71	7	71	371	1	544.44	22.50	3.07	9.89	12.00	40.67	45.00	80.00
3	777	72	7	71	372	1	935.55	20.00	3.48	32.25	11.67	46.33	50.00	88.00
3	778	73	7	71	373	1	887.78	33.90	2.64	6.67	11.55	30.00	30.00	73.00
3	779	74	7	71	374	1	136.67	17.00	2.48	14.50	11.00	41.33	40.00	80.00
3	780	75	7	71	375	1	1780.00	27.20	2.67	22.38	15.50	46.67	40.00	80.00
3	781	82	7	71	382	1	1708.89	43.50	2.69	7.78	14.80	51.67	40.00	73.00
3	782	83	7	72	383	1	1691.11	41.00	2.84	11.82	13.00	44.33	35.00	80.00
3	783	85	7	72	385	1	444.44	21.80	2.71	13.00	13.40	52.00	50.00	88.00
3	784	89	7	72	389	1	531.11	39.20	2.03	5.08	11.00	40.00	35.00	80.00
3	785	91	7	72	391	1	2042.22	37.80	2.83	11.13	15.60	46.67	35.00	73.00
3	786	93	7	72	393	1	1704.44	39.20	3.88	5.00	11.00	40.00	35.00	73.00
3	787	94	7	72	394	1	495.56	19.40	2.01	6.50	12.40	50.67	50.00	88.00
3	788	95	7	72	395	1	790.11	11.80	2.22	6.43	12.00	53.33	50.00	80.00
3	789	97	7	72	397	1	1933.33	36.20	2.60	7.91	12.80	48.67	40.00	73.00
3	790	98	7	72	398	1	1513.33	29.80	3.36	7.67	13.40	48.67	40.00	73.00
3	791	99	7	72	399	1	2108.89	31.80	2.75	10.10	11.20	50.33	40.00	73.00
3	792	100	7	72	3100	1	324.44	16.40	2.49	6.38	14.50	53.67	50.00	80.00
3	793	101	7	73	3101	1	1726.66	35.80	3.12	7.56	14.20	45.00	40.00	73.00
3	794	102	7	73	3102	1	1457.78	32.60	3.22	7.06	10.80	47.33	35.00	73.00
3	795	104	7	73	3104	1	1173.33	39.80	2.82	5.68	13.00	50.00	40.00	80.00
3	796	105	7	73	3105	1	1046.67	19.00	2.66	11.89	13.60	51.67	50.00	80.00
3	797	107	7	73	3107	1	1073.33	31.80	3.00	7.00	13.40	47.67	35.00	73.00
3	798	110	7	73	3110	1	1355.55	40.20	3.40	7.67	11.20	52.67	40.00	80.00
3	799	111	7	73	3111	1	1353.33	36.60	3.25	12.75	15.20	46.33	40.00	73.00

Year	Plot	Acessions	Replication	Block	Interaction	Observation /Plot	YDSD	SW	SDPD	PDPL	FPIH	PLHT	DF	DPM
3	800	112	7	73	3112	1	1786.66	23.60	3.29	17.08	13.60	49.33	45.00	80.00
3	801	113	7	73	3113	1	3146.66	23.00	4.13	16.25	16.00	61.33	50.00	80.00
3	802	114	7	73	3114	1	677.78	33.20	1.99	4.45	9.80	50.55	35.00	88.00
3	803	115	7	73	3115	1	782.22	37.40	1.98	5.81	10.40	36.67	35.00	73.00
3	804	117	7	74	3117	1	473.33	17.00	1.81	17.00	15.66	49.33	50.00	88.00
3	805	119	7	74	3119	1	1293.33	43.20	2.11	6.50	11.00	40.33	35.00	73.00
3	806	120	7	74	3120	1	1097.78	26.80	2.44	15.33	12.00	49.00	40.00	80.00
3	807	121	7	74	3121	1	1980.00	20.60	3.34	37.25	11.60	51.00	40.00	88.00
3	808	128	7	74	3128	1	1348.89	36.80	2.74	8.00	13.80	51.67	40.00	73.00
3	809	138	7	74	3138	1	1588.89	24.00	3.65	13.60	13.60	53.33	50.00	88.00
3	810	139	7	74	3139	1	1090.00	35.60	2.11	4.50	13.60	46.33	35.00	80.00
3	811	144	7	74	3144	1	700.66	15.80	1.78	7.73	13.40	50.00	50.00	88.00
3	812	153	7	74	3153	1	1013.33	20.20	3.15	19.75	14.00	50.00	50.00	88.00
3	813	154	7	74	3154	1	980.74	36.30	2.60	5.00	13.40	49.00	35.00	73.00
3	814	156	7	74	3156	1	472.22	20.90	3.46	10.14	13.00	50.67	50.00	80.00
3	815	161	7	75	3161	1	1780.00	40.40	2.93	14.20	14.60	52.00	45.00	80.00
3	816	162	7	75	3162	1	360.00	27.20	1.95	5.91	10.60	42.33	35.00	88.00
3	817	163	7	75	3163	1	380.56	35.87	2.10	5.88	10.00	41.33	35.00	80.00
3	818	164	7	75	3164	1	691.11	20.80	2.43	5.93	12.00	45.33	40.00	88.00
3	819	165	7	75	3165	1	1102.22	38.80	2.46	6.44	11.20	57.67	40.00	80.00
3	820	166	7	75	3166	1	200.56	32.00	2.00	3.00	12.00	42.33	35.00	88.00
3	821	167	7	75	3167	1	242.22	54.50	2.00	5.00	12.50	39.67	35.00	88.00
3	822	168	7	75	3168	1	1100.11	20.60	2.86	23.40	12.55	59.00	50.00	88.00
3	823	169	7	75	3169	1	1108.89	24.60	2.77	20.44	10.00	40.00	45.00	80.00
3	824	170	7	75	3170	1	1691.11	23.80	3.77	12.67	14.80	50.33	50.00	88.00
3	825	172	7	75	3172	1	1244.44	43.80	2.72	7.57	10.80	37.67	35.00	73.00
3	826	174	7	76	3174	1	1635.00	34.00	2.22	5.31	13.60	46.33	45.00	80.00
3	827	175	7	76	3175	1	1626.67	38.00	2.52	10.47	16.00	47.33	40.00	80.00
3	828	188	7	76	3188	1	1054.44	39.40	2.94	4.39	9.60	54.00	40.00	73.00
3	829	191	7	76	3191	1	1222.22	41.40	2.70	6.41	13.00	49.67	40.00	88.00
3	830	194	7	76	3194	1	1257.67	42.00	2.80	3.85	13.20	49.67	40.00	73.00
3	831	197	7	76	3197	1	894.44	36.40	1.79	6.00	12.80	45.67	40.00	80.00

Year	Plot	Acessions	Replication	Block	Interaction	Observation /Plot	YDSD	SW	SDPD	PDPL	FPIH	PLHT	DF	DPM
3	832	199	7	76	3199	1	693.33	24.40	2.67	10.92	12.20	41.33	45.00	80.00
3	833	200	7	76	3200	1	2951.11	20.60	3.59	26.33	15.00	51.67	50.00	88.00
3	834	201	7	76	3201	1	826.67	27.40	3.55	15.20	13.00	49.67	50.00	88.00
3	835	205	7	76	3205	1	501.94	16.60	3.00	9.00	14.66	51.33	50.00	80.00
3	836	500	7	76	3500	1	443.33	21.30	3.20	6.27	14.80	48.67	50.00	80.00
3	837	501	7	77	3501	1	1861.00	19.60	1.67	11.70	14.66	52.67	50.00	88.00
3	838	502	7	77	3502	1	1824.44	22.40	4.09	22.00	13.00	58.00	50.00	88.00
3	839	503	7	77	3503	1	1426.67	26.40	3.51	7.60	15.40	48.00	45.00	80.00
3	840	504	7	77	3504	1	1022.22	25.40	3.79	20.67	15.67	51.00	50.00	88.00
3	841	505	7	77	3505	1	379.80	19.00	2.50	10.00	14.90	48.33	50.00	88.00
3	842	506	7	77	3506	1	351.11	35.30	1.96	5.88	14.00	50.67	40.00	80.00
3	843	507	7	77	3507	1	356.88	18.00	4.00	9.44	13.10	50.00	50.00	88.00
3	844	508	7	77	3508	1	1017.78	33.80	2.93	8.91	15.20	50.33	35.00	73.00
3	845	509	7	77	3509	1	790.23	22.00	2.23	12.00	14.56	42.67	50.00	88.00
3	846	510	7	77	3510	1	1111.11	37.00	2.52	11.60	13.60	49.00	40.00	80.00
3	847	511	7	77	3511	1	122.22	11.00	2.50	5.71	15.00	50.00	50.00	88.00
3	848	1	8	78	31	1	320.00	20.10	2.03	11.29	12.40	40.00	39.00	73.00
3	849	3	8	78	33	1	1420.00	35.60	3.50	12.88	13.40	51.00	34.00	73.00
3	850	5	8	78	35	1	815.55	24.70	2.85	8.36	11.00	40.67	40.00	80.00
3	851	6	8	78	36	1	845.55	40.00	2.77	10.33	13.00	40.00	32.00	73.00
3	852	11	8	78	311	1	842.22	40.60	2.20	4.60	11.40	38.00	33.00	73.00
3	853	12	8	78	312	1	1960.00	22.00	3.84	14.56	14.60	57.33	45.00	80.00
3	854	13	8	78	313	1	120.55	19.66	2.50	6.70	15.00	56.00	60.00	90.00
3	855	15	8	78	315	1	1428.89	33.70	3.00	8.13	14.80	50.67	40.00	73.00
3	856	20	8	78	320	1	608.89	34.40	2.53	6.33	14.20	50.00	40.00	80.00
3	857	22	8	78	322	1	1224.44	20.20	2.38	12.10	12.80	50.00	38.00	80.00
3	858	23	8	78	323	1	2671.11	27.80	2.38	13.58	21.20	58.67	49.00	88.00
3	859	24	8	79	324	1	1377.78	22.60	3.70	13.00	12.00	49.33	50.00	88.00
3	860	25	8	79	325	1	735.55	22.00	2.14	12.36	12.00	55.00	40.00	88.00
3	861	26	8	79	326	1	2884.44	25.60	3.80	16.32	12.60	51.67	50.00	80.00
3	862	27	8	79	327	1	2100.00	28.80	2.37	18.87	15.20	49.67	50.00	88.00
3	863	28	8	79	328	1	572.22	22.30	3.73	18.50	10.50	48.67	50.00	88.00

Year	Plot	Acessions	Replication	Block	Interaction	Observation /Plot	YDSD	SW	SDPD	PDPL	FPIH	PLHT	DF	DPM
3	864	29	8	79	329	1	815.55	20.50	3.25	34.25	10.50	45.00	50.00	88.00
3	865	30	8	79	330	1	1657.78	29.00	2.45	11.00	15.00	53.67	50.00	88.00
3	866	32	8	79	332	1	1610.00	20.30	3.01	25.25	13.40	53.67	50.00	88.00
3	867	33	8	79	333	1	1520.00	21.60	4.11	14.00	14.20	57.33	40.00	88.00
3	868	34	8	79	334	1	937.78	20.20	1.48	33.45	15.80	47.67	48.00	88.00
3	869	35	8	79	335	1	202.22	12.40	1.54	12.00	12.80	62.33	50.00	88.00
3	870	36	8	80	336	1	518.89	17.00	3.18	11.50	11.80	36.67	45.00	80.00
3	871	37	8	80	337	1	1282.22	35.00	2.10	8.25	11.00	46.00	40.00	73.00
3	872	38	8	80	338	1	1833.33	37.40	3.03	11.00	12.60	40.00	30.00	73.00
3	873	39	8	80	339	1	1204.44	28.00	2.78	6.65	9.00	41.05	32.00	73.00
3	874	40	8	80	340	1	1477.78	41.40	2.65	10.33	10.80	46.33	35.00	73.00
3	875	41	8	80	341	1	1404.44	36.00	2.70	9.00	8.80	40.00	35.00	73.00
3	876	42	8	80	342	1	1228.89	36.60	3.23	6.57	15.40	49.00	40.00	73.00
3	877	43	8	80	343	1	1464.44	30.00	3.11	6.60	10.00	40.00	38.00	73.00
3	878	44	8	80	344	1	407.78	20.20	3.10	12.00	11.40	41.33	40.00	88.00
3	879	45	8	80	345	1	503.33	16.20	2.24	8.73	16.20	50.00	50.00	88.00
3	880	46	8	80	346	1	535.56	16.20	2.95	7.31	11.20	39.33	40.00	73.00
3	881	47	8	81	347	1	262.22	16.40	4.44	3.60	12.50	43.00	50.00	80.00
3	882	49	8	81	349	1	272.22	16.50	2.00	14.83	18.00	49.00	50.00	88.00
3	883	52	8	81	352	1	1955.55	28.80	3.65	15.62	13.00	49.00	40.00	88.00
3	884	56	8	81	356	1	1266.67	24.10	3.17	11.27	11.40	43.67	40.00	73.00
3	885	57	8	81	357	1	737.78	22.20	2.55	9.24	13.20	54.00	50.00	88.00
3	886	58	8	81	358	1	385.56	24.50	1.38	12.00	13.20	42.00	50.00	88.00
3	887	60	8	81	360	1	566.66	15.60	2.00	6.00	13.56	50.33	50.00	88.00
3	888	61	8	81	361	1	1641.11	33.00	3.03	7.90	13.20	42.67	35.00	73.00
3	889	62	8	81	362	1	760.00	41.60	2.39	9.38	12.40	46.33	30.00	73.00
3	890	63	8	81	363	1	881.11	22.50	4.33	11.13	11.80	44.67	50.00	80.00
3	891	64	8	81	364	1	1791.11	34.20	3.34	6.35	12.40	50.00	40.00	73.00
3	892	65	8	82	365	1	1515.55	37.50	2.78	11.25	15.20	48.33	40.00	80.00
3	893	66	8	82	366	1	453.33	15.00	2.05	16.63	12.00	47.67	50.00	88.00
3	894	67	8	82	367	1	1495.55	37.60	2.09	11.64	10.40	34.33	30.00	73.00
3	895	69	8	82	369	1	810.44	18.20	2.33	7.17	12.00	37.00	50.00	88.00

Year	Plot	Acessions	Replication	Block	Interaction	Observation /Plot	YDSD	SW	SDPD	PDPL	FPIH	PLHT	DF	DPM
3	896	70	8	82	370	1	1515.55	35.60	2.51	14.00	13.80	47.67	40.00	80.00
3	897	71	8	82	371	1	864.44	23.60	2.95	9.93	13.60	47.00	45.00	80.00
3	898	72	8	82	372	1	500.31	16.55	3.26	9.50	10.22	44.00	50.00	80.00
3	899	73	8	82	373	1	1614.44	41.70	2.88	10.23	12.00	42.33	30.00	73.00
3	900	74	8	82	374	1	398.75	20.88	2.55	9.50	10.44	40.00	40.00	80.00
3	901	75	8	82	375	1	996.67	23.50	3.52	14.14	15.00	41.33	40.00	80.00
3	902	82	8	82	382	1	1306.00	36.70	1.93	20.50	14.00	50.67	40.00	88.00
3	903	83	8	83	383	1	1862.22	41.60	2.82	9.81	15.20	50.00	35.00	80.00
3	904	85	8	83	385	1	1353.33	23.20	2.95	13.94	17.80	48.00	50.00	88.00
3	905	89	8	83	389	1	1368.89	42.00	2.38	12.50	12.20	43.67	35.00	73.00
3	906	91	8	83	391	1	1327.78	36.60	3.11	7.93	11.80	47.33	35.00	73.00
3	907	93	8	83	393	1	1234.44	35.30	2.71	7.67	10.80	42.55	35.00	73.00
3	908	94	8	83	394	1	540.00	17.60	2.48	9.43	15.20	51.00	50.00	80.00
3	909	95	8	83	395	1	1308.89	20.80	3.75	27.29	12.80	47.67	50.00	88.00
3	910	97	8	83	397	1	2021.11	39.10	2.65	9.86	12.00	49.00	40.00	73.00
3	911	98	8	83	398	1	1640.00	32.60	2.82	11.50	13.20	42.33	40.00	73.00
3	912	99	8	83	399	1	1829.55	25.70	2.40	6.50	11.80	50.55	40.00	73.00
3	913	100	8	83	3100	1	486.00	17.40	1.19	7.00	14.22	50.67	50.00	80.00
3	914	101	8	84	3101	1	2015.55	35.60	3.16	8.37	12.60	46.00	40.00	73.00
3	915	102	8	84	3102	1	1224.44	33.20	3.70	9.90	12.00	40.67	35.00	73.00
3	916	104	8	84	3104	1	1491.11	33.00	2.94	7.71	10.60	55.00	40.00	73.00
3	917	105	8	84	3105	1	1160.33	18.40	4.00	5.80	12.00	49.33	50.00	88.00
3	918	107	8	84	3107	1	922.22	30.60	2.98	5.63	12.20	44.67	35.00	73.00
3	919	110	8	84	3110	1	1287.78	33.10	2.95	6.17	12.40	41.67	40.00	73.00
3	920	111	8	84	3111	1	1217.78	35.00	2.65	5.81	14.80	45.67	40.00	73.00
3	921	112	8	84	3112	1	2428.89	25.20	3.23	19.38	13.80	43.33	45.00	80.00
3	922	113	8	84	3113	1	2300.76	19.40	3.09	6.17	12.50	58.33	50.00	80.00
3	923	114	8	84	3114	1	704.44	33.00	3.06	3.32	10.00	49.66	35.00	88.00
3	924	115	8	84	3115	1	1368.89	37.60	2.87	5.84	13.60	44.33	35.00	73.00
3	925	117	8	85	3117	1	584.44	14.40	3.20	9.64	16.00	51.33	50.00	88.00
3	926	119	8	85	3119	1	1842.22	42.20	2.35	14.62	12.40	41.67	35.00	73.00
3	927	120	8	85	3120	1	2613.33	35.00	2.85	16.69	13.40	48.67	40.00	73.00

Year	Plot	Acessions	Replication	Block	Interaction	Observation /Plot	YDSD	SW	SDPD	PDPL	FPIH	PLHT	DF	DPM
3	928	121	8	85	3121	1	1082.22	18.80	2.31	14.20	13.80	47.00	40.00	88.00
3	929	128	8	85	3128	1	1373.33	35.30	3.25	7.79	13.60	50.00	40.00	73.00
3	930	138	8	85	3138	1	1508.89	21.80	3.21	10.40	12.80	51.33	50.00	80.00
3	931	139	8	85	3139	1	620.00	36.20	2.53	13.75	12.60	40.00	35.00	73.00
3	932	144	8	85	3144	1	653.97	16.60	2.43	4.00	12.00	45.67	50.00	80.00
3	933	153	8	85	3153	1	1004.44	17.70	2.77	16.90	14.60	44.67	50.00	88.00
3	934	154	8	85	3154	1	1061.11	30.50	3.07	6.18	10.60	50.54	35.00	73.00
3	935	156	8	85	3156	1	304.44	17.80	2.92	5.82	11.50	45.33	50.00	80.00
3	936	161	8	86	3161	1	518.89	34.60	2.40	6.67	14.60	46.67	45.00	80.00
3	937	162	8	86	3162	1	946.67	37.20	2.09	6.44	13.20	35.67	35.00	80.00
3	938	163	8	86	3163	1	226.67	32.80	1.66	4.75	11.55	45.00	35.00	80.00
3	939	164	8	86	3164	1	1022.22	23.50	2.57	9.15	14.40	47.33	40.00	80.00
3	940	165	8	86	3165	1	595.55	34.50	2.00	6.36	14.40	59.33	40.00	80.00
3	941	166	8	86	3166	1	150.99	36.80	2.10	3.80	11.44	40.00	35.00	88.00
3	942	167	8	86	3167	1	331.11	39.70	1.70	3.14	10.80	37.00	35.00	88.00
3	943	168	8	86	3168	1	1748.89	22.40	3.31	13.33	13.60	52.33	50.00	88.00
3	944	169	8	86	3169	1	1010.12	21.40	2.80	10.13	10.00	42.33	45.00	80.00
3	945	170	8	86	3170	1	1573.33	24.40	4.08	13.93	15.00	53.67	50.00	88.00
3	946	172	8	86	3172	1	983.33	42.00	2.48	8.50	11.20	40.12	35.00	73.00
3	947	174	8	87	3174	1	1880.00	37.20	3.08	7.89	14.00	50.00	45.00	80.00
3	948	175	8	87	3175	1	890.00	34.00	2.00	7.87	15.00	49.33	40.00	80.00
3	949	188	8	87	3188	1	1003.33	35.60	3.00	4.76	13.00	46.00	40.00	73.00
3	950	191	8	87	3191	1	1080.55	35.00	2.50	5.55	11.66	50.00	40.00	88.00
3	951	194	8	87	3194	1	1126.67	36.00	3.21	7.64	12.00	40.67	40.00	73.00
3	952	197	8	87	3197	1	713.33	30.60	2.26	6.00	15.60	50.00	40.00	80.00
3	953	199	8	87	3199	1	493.33	22.20	3.60	10.83	12.00	42.33	45.00	88.00
3	954	200	8	87	3200	1	1822.22	14.10	2.11	10.77	12.60	42.33	50.00	88.00
3	955	201	8	87	3201	1	1632.22	24.30	3.19	20.64	14.60	46.67	50.00	88.00
3	956	205	8	87	3205	1	341.11	15.30	2.04	17.00	14.00	50.00	50.00	88.00
3	957	500	8	87	3500	1	100.00	15.00	1.88	3.20	10.40	45.00	50.00	80.00
3	958	501	8	88	3501	1	2377.78	25.20	2.84	25.38	16.20	54.33	50.00	88.00
3	959	502	8	88	3502	1	1045.55	22.30	2.68	18.82	14.80	59.67	50.00	88.00

Year	Plot	Acessions	Replication	Block	Interaction	Observation /Plot	YDSD	SW	SDPD	PDPL	FPIH	PLHT	DF	DPM
3	960	503	8	88	3503	1	1787.78	30.40	2.81	7.50	14.60	52.33	45.00	80.00
3	961	504	8	88	3504	1	1377.78	24.20	3.90	17.30	14.80	51.67	50.00	88.00
3	962	505	8	88	3505	1	595.55	18.60	2.72	19.86	12.80	52.67	50.00	88.00
3	963	506	8	88	3506	1	1385.55	44.20	1.98	8.40	12.80	44.33	40.00	80.00
3	964	507	8	88	3507	1	294.44	15.30	2.38	16.67	12.00	42.00	50.00	88.00
3	965	508	8	88	3508	1	1654.44	32.60	2.53	10.06	11.80	51.67	35.00	73.00
3	966	509	8	88	3509	1	566.44	21.20	3.40	10.50	16.40	50.33	50.00	88.00
3	967	510	8	88	3510	1	1414.44	33.70	3.99	6.67	11.80	49.00	40.00	80.00
3	968	511	8	88	3511	1	345.65	14.10	2.90	8.56	12.65	42.33	50.00	88.00
3	969	1	9	89	31	1	617.78	20.00	2.31	12.40	11.20	40.00	39.00	73.00
3	970	3	9	89	33	1	1162.22	38.60	2.84	11.88	13.20	50.67	34.00	80.00
3	971	5	9	89	35	1	750.33	16.70	1.76	4.10	12.00	41.67	41.00	88.00
3	972	6	9	89	36	1	836.67	35.90	1.99	6.75	10.80	43.00	32.00	73.00
3	973	11	9	89	311	1	861.11	39.00	2.21	5.60	12.40	37.00	33.00	73.00
3	974	12	9	89	312	1	1717.78	25.60	3.38	13.38	15.00	56.33	45.00	80.00
3	975	13	9	89	313	1	135.56	14.00	1.38	9.00	18.60	57.67	60.00	88.00
3	976	15	9	89	315	1	1130.44	24.40	1.97	7.78	11.20	53.33	40.00	80.00
3	977	20	9	89	320	1	866.67	36.30	1.87	6.24	12.20	50.67	40.00	80.00
3	978	22	9	89	322	1	1402.22	27.60	3.30	14.00	12.40	50.00	38.00	80.00
3	979	23	9	89	323	1	2082.66	29.40	2.27	10.00	16.40	56.33	49.00	88.00
3	980	24	9	90	324	1	1460.44	24.00	3.36	10.75	14.40	49.00	50.00	88.00
3	981	25	9	90	325	1	1052.22	23.90	3.21	14.00	14.20	51.33	40.00	88.00
3	982	26	9	90	326	1	1683.33	24.40	1.71	20.75	14.40	50.00	50.00	80.00
3	983	27	9	90	327	1	584.44	28.80	2.27	8.30	13.80	64.67	50.00	88.00
3	984	28	9	90	328	1	532.22	18.80	2.90	19.20	15.60	45.00	50.00	88.00
3	985	29	9	90	329	1	622.22	22.50	3.78	9.67	11.00	50.00	50.00	88.00
3	986	30	9	90	330	1	1100.00	30.00	2.73	7.89	13.60	52.33	50.00	88.00
3	987	32	9	90	332	1	1608.89	17.70	3.90	16.80	12.60	53.33	50.00	88.00
3	988	33	9	90	333	1	1437.78	18.90	1.72	9.75	15.00	59.33	40.00	88.00
3	989	34	9	90	334	1	337.78	15.40	1.33	23.40	15.00	62.33	48.00	88.00
3	990	35	9	90	335	1	193.33	11.50	1.25	29.20	13.90	56.67	50.00	88.00
3	991	36	9	91	336	1	478.89	17.20	1.86	13.33	17.60	40.67	45.00	88.00

Year	Plot	Acessions	Replication	Block	Interaction	Observation /Plot	YDSD	SW	SDPD	PDPL	FPIH	PLHT	DF	DPM
3	992	37	9	91	337	1	2226.66	34.90	3.19	10.59	14.80	42.33	40.00	73.00
3	993	38	9	91	338	1	931.11	38.90	1.83	8.93	11.60	40.00	30.00	73.00
3	994	39	9	91	339	1	1738.89	31.90	3.96	9.40	13.00	40.00	32.00	73.00
3	995	40	9	91	340	1	1797.78	40.00	2.93	8.69	14.60	46.67	35.00	73.00
3	996	41	9	91	341	1	817.78	41.40	1.80	12.22	10.40	37.00	35.00	73.00
3	997	42	9	91	342	1	1191.11	43.20	3.28	4.16	15.20	42.00	40.00	73.00
3	998	43	9	91	343	1	1946.66	32.00	2.88	14.69	12.00	40.00	38.00	73.00
3	999	44	9	91	344	1	791.11	19.60	3.28	14.88	13.80	52.00	40.00	80.00
3	1000	45	9	91	345	1	648.50	11.70	2.00	4.29	12.55	55.33	50.00	88.00
3	1001	46	9	91	346	1	1512.22	21.30	3.05	12.89	12.80	48.00	40.00	73.00
3	1002	47	9	92	347	1	125.56	14.30	2.32	4.25	12.00	51.00	50.00	80.00
3	1003	49	9	92	349	1	420.50	18.89	2.10	11.66	15.00	36.67	50.00	88.00
3	1004	52	9	92	352	1	1044.44	27.20	2.68	20.88	11.00	42.00	40.00	88.00
3	1005	56	9	92	356	1	1131.11	23.80	3.30	7.26	12.60	47.00	40.00	73.00
3	1006	57	9	92	357	1	585.55	25.90	2.30	10.33	12.20	52.67	50.00	80.00
3	1007	58	9	92	358	1	418.99	22.45	2.00	9.55	12.56	45.00	50.00	88.00
3	1008	60	9	92	360	1	630.00	20.30	1.71	12.82	12.40	50.67	50.00	88.00
3	1009	61	9	92	361	1	1132.22	35.00	3.02	5.16	12.60	50.67	35.00	80.00
3	1010	62	9	92	362	1	1721.11	39.80	2.75	9.27	13.20	46.33	30.00	73.00
3	1011	63	9	92	363	1	654.44	18.50	3.67	8.91	13.20	50.00	50.00	80.00
3	1012	64	9	92	364	1	1627.50	24.50	1.94	4.17	13.80	50.67	40.00	73.00
3	1013	65	9	93	365	1	1668.50	30.10	2.50	6.06	15.80	47.67	40.00	88.00
3	1014	66	9	93	366	1	743.33	18.90	2.67	9.28	14.60	50.00	50.00	88.00
3	1015	67	9	93	367	1	557.78	30.10	1.98	5.50	12.20	37.00	30.00	73.00
3	1016	69	9	93	369	1	1022.22	25.20	3.33	11.69	14.40	53.67	50.00	88.00
3	1017	70	9	93	370	1	1735.55	38.20	2.85	7.89	13.00	50.00	40.00	80.00
3	1018	71	9	93	371	1	700.86	19.00	2.71	8.29	9.40	51.67	45.00	73.00
3	1019	72	9	93	372	1	128.89	13.18	0.93	10.56	12.00	48.67	50.00	80.00
3	1020	73	9	93	373	1	552.22	33.80	2.11	5.85	10.60	35.00	30.00	73.00
3	1021	74	9	93	374	1	648.89	21.50	3.43	5.00	11.00	38.00	40.00	80.00
3	1022	75	9	93	375	1	1850.00	26.60	3.60	12.94	15.00	47.00	40.00	73.00
3	1023	82	9	93	382	1	905.55	37.20	1.68	7.50	14.20	51.33	40.00	73.00

Year	Plot	Acessions	Replication	Block	Interaction	Observation /Plot	YDSD	SW	SDPD	PDPL	FPIH	PLHT	DF	DPM
3	1024	83	9	94	383	1	1848.89	39.80	2.73	7.71	14.00	40.67	35.00	73.00
3	1025	85	9	94	385	1	898.55	22.66	2.64	13.55	15.88	54.00	50.00	88.00
3	1026	89	9	94	389	1	680.00	40.40	1.48	9.33	9.60	46.00	35.00	80.00
3	1027	91	9	94	391	1	784.44	35.10	2.21	5.63	14.60	50.00	35.00	80.00
3	1028	93	9	94	393	1	1082.22	38.80	3.43	7.00	13.00	38.00	35.00	73.00
3	1029	94	9	94	394	1	457.78	18.60	2.84	12.75	13.20	57.00	50.00	88.00
3	1030	95	9	94	395	1	280.00	14.90	2.64	6.73	11.80	55.00	50.00	88.00
3	1031	97	9	94	397	1	1965.00	39.20	1.19	7.00	9.20	55.67	40.00	88.00
3	1032	98	9	94	398	1	1550.00	32.40	3.10	9.71	13.80	50.00	40.00	73.00
3	1033	99	9	94	399	1	1553.33	30.60	3.01	7.85	10.60	50.00	40.00	73.00
3	1034	100	9	94	3100	1	648.89	21.80	2.50	6.17	14.60	53.00	50.00	88.00
3	1035	101	9	95	3101	1	1972.22	33.60	3.82	9.07	14.60	45.00	40.00	73.00
3	1036	102	9	95	3102	1	833.33	34.40	2.74	6.17	13.00	50.00	35.00	80.00
3	1037	104	9	95	3104	1	1200.55	33.60	1.32	6.71	13.80	40.00	40.00	80.00
3	1038	105	9	95	3105	1	1288.89	20.00	3.86	12.50	15.00	51.67	50.00	88.00
3	1039	107	9	95	3107	1	634.44	34.60	2.86	7.22	13.40	41.33	35.00	73.00
3	1040	110	9	95	3110	1	1935.55	34.60	3.51	7.58	12.00	40.67	40.00	73.00
3	1041	111	9	95	3111	1	1654.44	35.50	2.69	7.43	12.20	51.33	40.00	73.00
3	1042	112	9	95	3112	1	1462.22	27.60	2.95	11.40	13.60	55.67	45.00	80.00
3	1043	113	9	95	3113	1	1651.11	21.80	2.94	13.37	16.00	51.00	50.00	80.00
3	1044	114	9	95	3114	1	662.22	36.80	1.45	6.65	17.60	40.00	35.00	88.00
3	1045	115	9	95	3115	1	945.55	42.20	2.27	5.50	12.80	40.00	35.00	80.00
3	1046	117	9	96	3117	1	618.89	15.70	2.46	12.13	17.00	50.00	50.00	88.00
3	1047	119	9	96	3119	1	2420.00	41.30	2.50	11.20	11.80	44.33	35.00	73.00
3	1048	120	9	96	3120	1	802.22	30.00	2.79	20.29	12.40	50.00	40.00	73.00
3	1049	121	9	96	3121	1	1040.58	20.55	2.69	25.55	12.44	50.00	40.00	88.00
3	1050	128	9	96	3128	1	1144.44	40.60	3.27	5.64	12.80	44.00	40.00	80.00
3	1051	138	9	96	3138	1	1480.63	20.80	2.67	8.85	12.40	52.66	50.00	88.00
3	1052	139	9	96	3139	1	1560.00	44.00	3.60	6.47	13.80	46.33	35.00	88.00
3	1053	144	9	96	3144	1	1102.22	21.60	2.87	9.21	17.20	50.00	50.00	88.00
3	1054	153	9	96	3153	1	1100.10	20.30	2.15	11.50	12.40	54.67	50.00	88.00
3	1055	154	9	96	3154	1	1022.22	30.40	3.09	5.82	9.80	51.00	35.00	73.00

Year	Plot	Acessions	Replication	Block	Interaction	Observation /Plot	YDSD	SW	SDPD	PDPL	FPIH	PLHT	DF	DPM
3	1056	156	9	96	3156	1	368.89	16.10	2.75	12.67	11.80	50.00	50.00	88.00
3	1057	161	9	97	3161	1	1100.00	25.60	2.35	6.67	14.55	50.00	45.00	80.00
3	1058	162	9	97	3162	1	345.56	30.50	1.72	5.00	12.40	48.00	35.00	80.00
3	1059	163	9	97	3163	1	553.33	45.00	2.19	6.33	12.60	50.00	35.00	80.00
3	1060	164	9	97	3164	1	685.55	20.90	2.81	8.50	11.80	41.67	40.00	80.00
3	1061	165	9	97	3165	1	850.55	32.55	2.50	6.20	12.55	48.33	40.00	80.00
3	1062	166	9	97	3166	1	228.77	35.55	1.80	3.10	10.88	40.00	35.00	88.00
3	1063	167	9	97	3167	1	250.45	40.55	2.00	4.10	11.55	38.00	35.00	88.00
3	1064	168	9	97	3168	1	724.44	19.40	2.85	13.70	13.20	52.67	50.00	88.00
3	1065	169	9	97	3169	1	1035.55	23.60	2.85	10.41	13.80	41.33	45.00	80.00
3	1066	170	9	97	3170	1	1351.11	22.00	4.11	12.64	14.40	58.00	50.00	88.00
3	1067	172	9	97	3172	1	1022.22	38.40	2.60	10.56	13.40	40.00	35.00	73.00
3	1068	174	9	98	3174	1	1633.33	34.80	2.85	8.89	15.80	49.33	45.00	80.00
3	1069	175	9	98	3175	1	1264.44	30.50	1.28	4.36	14.40	40.00	40.00	80.00
3	1070	188	9	98	3188	1	1391.11	38.00	3.46	7.92	11.00	45.00	40.00	73.00
3	1071	191	9	98	3191	1	875.66	34.40	1.53	5.22	12.40	52.67	40.00	88.00
3	1072	194	9	98	3194	1	1388.89	39.60	3.21	7.31	12.40	51.00	40.00	73.00
3	1073	197	9	98	3197	1	803.50	27.20	1.60	2.79	10.80	50.67	40.00	80.00
3	1074	199	9	98	3199	1	752.22	26.70	3.53	12.00	13.00	48.00	45.00	80.00
3	1075	200	9	98	3200	1	815.55	20.40	1.83	18.00	12.40	48.00	50.00	88.00
3	1076	201	9	98	3201	1	622.22	21.80	2.41	11.58	13.20	52.33	50.00	88.00
3	1077	205	9	98	3205	1	792.22	18.80	2.22	20.80	14.60	50.00	50.00	88.00
3	1078	500	9	98	3500	1	341.11	16.70	2.20	4.74	13.60	50.00	50.00	80.00
3	1079	501	9	99	3501	1	1345.55	24.40	1.69	17.80	15.80	56.00	50.00	88.00
3	1080	502	9	99	3502	1	1434.50	19.20	1.63	7.94	13.80	62.67	50.00	88.00
3	1081	503	9	99	3503	1	1605.50	26.00	2.03	5.67	16.00	50.33	45.00	88.00
3	1082	504	9	99	3504	1	937.78	20.60	2.56	12.00	15.40	53.67	50.00	88.00
3	1083	505	9	99	3505	1	439.66	20.00	3.00	14.66	15.00	55.00	50.00	88.00
3	1084	506	9	99	3506	1	888.89	35.00	1.59	3.15	13.20	50.00	40.00	80.00
3	1085	507	9	99	3507	1	401.99	17.20	3.50	9.66	14.55	52.33	50.00	88.00
3	1086	508	9	99	3508	1	1277.78	37.40	2.96	8.08	12.40	50.00	35.00	73.00
3	1087	509	9	99	3509	1	497.12	19.00	2.88	11.80	15.00	50.00	50.00	88.00

Year	Plot	Acessions	Replication	Block	Interaction	Observation /Plot	YDSD	SW	SDPD	PDPL	FPIH	PLHT	DF	DPM
3	1088	510	9	99	3510	1	1337.78	37.40	2.98	6.26	9.80	52.33	40.00	80.00
3	1089	511	9	99	3511	1	401.89	16.90	2.11	9.40	14.55	54.33	50.00	88.00

YDSD: grain yield, SW: 100-seed weight, SDPD: number of seeds per pod, PDPL: number of pods per plant, FPIH: first pod insertion height, PLHT: plant height, DF: number of days to flowering, and DPM: number of days to maturity.
