# Supplementary material for: Genome-Wide Association for Morphological and Agronomic Traits in Phaseolus vulgaris L. Accessions
Source: Plants (Basel). 2024 Sep 21;13(18):2638. doi: 10.3390/plants13182638 (PMC11435040; doi:10.3390/plants13182638)
Supplement: Supplementary file 1 [file plants-13-02638-s001.zip › Table S2.pdf]

Table S2 Accessions from Nupagri-UEM germoplasm bank, gene pool, origin, market class evaluated in the present study.

Acession ID	Acession name	Gene Pool[a]	Origin[b]	Market class
BGF 1	Rosinha PR	M	PR	Pink bean
BGF 5	Rosinha PR II	M	PR	Pink bean
BGF 12	Mesclado	M	PR	Pinto
BGF 13	Pixirum	M	PR	Cranberry
BGF 22	Carioca II	M	PR	Cranberry
BGF 23	Carioca III	M	PR	Cranberry
BGF 24	Carioca IV	M	PR	Cranberry
BGF 25	Carioca V	M	PR	Cranberry
BGF 26	Carioca VI	M	PR	Cranberry
BGF 27	Carioca Claro	M	PR	Cranberry
BGF 28	Carioca Pintado I	M	PR	Pinto
BGF 29	Carioca Pintado II	M	PR	Pinto
BGF 30	Carioca Pitoko	M	PR	Cranberry
BGF 32	Preto I	M	PR	Black
BGF 33	Preto II	M	PR	Black
BGF 34	Preto III	M	PR	Black
BGF 35	Preto IV	M	PR	Black
BGF 36	Rosinha Paraná	M	PR	Pink
BGF 44	Rosinha Opaco	M	MS	Pink
BGF 45	Rosinha A	M	MS	Pink
BGF 46	Rosinha B	M	MS	Pink
BGF 47	Rosinha C	M	MS	Pink
BGF 49	Roxinho A	M	MS	Light purple
BGF 52	Mulatão Lustroso	M	MS	Dark red
BGF 56	Mulatinho Vagem Roxa B	M	MS	Light brown
BGF 57	Carioca Vagem Rosada	M	MS	Cranberry
BGF 58	Rosinha	M	MS	Pink
BGF 60	Uberabinha Preto	M	MS	Black
BGF 63	Carioca sem Cipó	M	MS	Cranberry
BGF 66	Rosinha sem Cipó	M	MS	Pink
BGF 69	Carioca com Cipó	M	MS	Cranberry
BGF 71	Roxinho Mineiro	M	MS	Light purple
BGF 72	Preto Guamirim	M	MS	Black
BGF 74	Rosinha Guaicucos	M	MS	Pink
BGF 75	Cara Suja	M	MS	Dark brown
BGF 85	Preto SC	M	SC	Black
BGF 94	Crioulo Brilhoso	M	MS	Black
BGF 95	Crioulo 159	M	MS	Black
BGF 105	FC 117	M	SC	Black
BGF 112	Porto Real	M	MS	Cranberry
BGF 113	Safira	M	MS	Cranberry
BGF 117	SC 1	M	SC	Dark brown
BGF 121	Guarumbé	M	PR	Cranberry
BGF 138	Princesa	M	GO	Cranberry
BGF 144	BAT 93	M	MG	Light brown

Acession ID	Acession name	Gene Pool[a]	Origin[b]	Market class
BGF 153	Mãezinha	M	SC	Light brown
BGF 156	Carioca Novo	M	SC	Cranberry
BGF 164	Roxo Mineiro	M	PR	Light purple
BGF 168	Preto Argentino	M	PR	Black
BGF 169	Roxinho PR	M	PR	Light purple
BGF 170	Uberabinha PR	M	PR	Black
BGF 199	Feijão Cara Suja	M	PR	Dark brown
BGF 200	Feijão Rosinha	M	PR	Pink
BGF 201	Feijão Moro	M	PR	Dark brown
BGF 205	Rosa	M	PR	Pink
BGF 3	Preto Andino	A	PR	Black
BGF 6	Rajado	A	PR	Light brown kidney striped
BGF 11	Manteigão Rajado	A	PR	Light brown kidney striped
BGF 15	Pitanga	A	PR	Light brown kidney
BGF 20	Beija-Flor	A	PR	Light brown kidney
BGF 37	Roxinho Paraná	A	PR	Light purple
BGF 38	Jalo Listra Pretas	A	PR	Light brown kidney striped
BGF 39	Jalo Pardo	A	PR	Light brown kidney
BGF 40	Jalo Pintado I	A	PR	Light brown pink striped kidney
BGF 41	Jalo Pintado II	A	PR	Light brown pink striped kidney
BGF 42	Jalo Mulato	A	MS	Light brown kidney
BGF 43	Bolinha	A	MS	Light brown kidney striped
BGF 61	Manteiguinha de Cipó	A	MS	Light brown kidney
BGF 62	Jalo sem Cipó	A	MS	Light brown kidney
BGF 64	Bodoquena	A	MS	Light brown kidney
BGF 65	Chita Bonita	A	MS	Cranberry
BGF 67	Manteiga sem Cipó	A	MS	Light brown kidney
BGF 70	Bolinha	A	MS	Light brown kidney
BGF 73	Manteigão	A	MS	Light brown kidney
BGF 82	Carnaval 1 SC	A	SC	Light yellow round kidney
BGF 83	Carnaval 2 SC	A	SC	Light yellow round kidney
BGF 89	Carnaval Mix SC	A	SC	Light yellow round kidney
BGF 91	Preto Brilhoso Achatado	A	SC	Black
BGF 93	Crioulo Manteiga	A	SC	Black
BGF 97	FC 2016	A	SC	Black
BGF 98	FC 2001	A	SC	Black
BGF 99	FC 2045	A	SC	Black
BGF 100	Crioulo Ponte Serrada	A	SC	Black
BGF 101	FC 2063	A	SC	Black
BGF 102	FC 2010	A	SC	Black
BGF 104	CN 694 FC 1212	A	SC	Black
BGF 107	Preto Chatinho	A	SC	Black
BGF 110	Azulão Ponte Serrada	A	SC	Black
BGF 111	Azulão Ab. Luz	A	SC	Black
BGF 114	Amendoim Cavalo	A	SC	Light brown kidney
BGF 115	Vermelho Tozzo	A	SC	Red
BGF 119	Jalo Listras Vermelhas	A	PR	Cranberry

Acession ID	Acession name	Gene Pool[a]	Origin[b]	Market class
BGF 120	Rosa	A	PR	Pink
BGF 128	Jalo Precoce	A	PR	Light brown kidney
BGF 139	Jalo Vermelho	A	PR	Red kidney
BGF 154	Bolinha Rajado	A	PR	Cranberry
BGF 161	Bolinha Vermelho	A	PR	Red kidney
BGF 162	Bolinha Amendoim	A	PR	Red kidney
BGF 163	Branco PR	A	PR	White Kidney
BGF 165	Jalo B	A	PR	Light brown kidney
BGF 166	Branco Argentino	A	PR	White Kidney
BGF 167	Cavalo	A	PR	Light red kidney
BGF 172	Carnaval 1 PR	A	PR	Cranberry
BGF 174	Bolinha 1 PR	A	PR	Light yellow kidney
BGF 175	Bolinha 2 PR	A	PR	Light brown kidney
BGF 188	Jalo A	A	PR	Light brown kidney
BGF 191	Jalo BR	A	PR	Light brown kidney
BGF 194	Jalo	A	PR	Light brown kidney
BGF 197	Jalo EEP558	A	MG	Light brown kidney

[a] Gene Pool: A Andean, M Mesoamerican; [b]: States in Brazil where common beans were coleted: MG, Minas Gerais; MS, Mato Grosso do Sul; PR, Paraná; SC, Santa Catarina.
